# Toward ultimate NMR resolution with deep learning

**DOI:** 10.1126/sciadv.ady7995

**Published:** 2026-03-27

**Authors:** Amir Jahangiri, Tatiana Agback, Ulrika Brath, Vladislav Orekhov

**Affiliations:** ^1^ Department of Chemistry and Molecular Biology, University of Gothenburg, Gothenburg 40530, Sweden.; ^2^ Department of Molecular Sciences, Swedish University of Agricultural Sciences, Uppsala 75007, Sweden.; ^3^ Swedish NMR Centre, SciLifeLab, University of Gothenburg, P.O. Box 465, Gothenburg 40530, Sweden.

## Abstract

Resolution in NMR is defined as the ability to distinguish and accurately determine signal positions while mitigating overlap. In the pursuit of ultimate resolution, we introduce peak probability presentations (*P*^3^), a statistical spectral representation that assigns a probability to each spectral point, indicating the likelihood that a peak maximum occurs at that location. The mapping between the traditional spectrum and *P*^3^ is achieved using MR-Ai, a physics-inspired and computationally efficient deep-learning neural network. *P*^3^ is validated on 60 database proteins and showcased on the challenging Tau and MATL1 proteins. Using synthetic spectra, we show that the achieved peak-localization precision closely approaches the theoretical limits set by the Cramér-Rao lower bound and Bayesian Monte Carlo estimates. Furthermore, MR-Ai enables the coprocessing of multiple spectra, facilitating direct information exchange between datasets to enhance spectral quality, particularly in cases of highly sparse sampling.

## Teaser

Deep learning delivers super-resolution for multidimensional NMR spectra.

## INTRODUCTION

Nuclear magnetic resonance (NMR) spectroscopy has provided increasingly valuable insights into the behavior and properties of molecules. Over the past decades, it has established itself as an essential atomic-level tool in structural biology (*1*) and has played a crucial role in protein structural analysis (*2*). Despite its versatility, the NMR spectroscopy of biological systems is often constrained by limited spectral resolution, where signal overlap complicates chemical shift assignment and interferes with the analysis of molecular dynamics and structure (*3*). Since the introduction of Fourier NMR spectroscopy in the mid-1960s (*4*), numerous signal processing methods have been developed and are now routinely used to enhance resolution. These include traditional techniques such as zero padding (*5*, *6*), apodization with weighting functions (*7*), linear prediction (*8*), and virtual decoupling through deconvolution (*9*–*12*). In response to the limitations of traditional approaches, artificial intelligence (AI), particularly deep learning (DL), has demonstrated substantial potential across various areas of NMR research (*13*–*15*). DL-based tools not only surpass traditional NMR techniques in rapid and high-quality nonuniformly sampled (NUS) reconstruction (*16*–*19*); efficient homonuclear decoupling (*19*, *20*), pure shift spectra generation (*21*–*23*); spectra denoising (*24*, *25*); automated peak picking (*26*–*28*); and, more recently, spectral signal sharpening (*29*) but also have the potential to extend beyond the boundaries of traditional magnetic resonance processing, for example, enabling reference-free assessment of the spectra quality and obtaining phase sensitive spectra without quadrature detection (*30*).

In this work, we address the challenge of achieving ultimate spectral resolution and reducing data complexity in experimental NMR spectra, which are limited by discrete Fourier transform (DFT), thermal noise, spectral artifacts, and signal overlap. We consider two complementary aspects of the “resolution” concept. First, in the formal statistical sense, resolution refers to the inherent precision with which peak positions (and other spectral parameters) can be estimated from the recorded time-domain data given an explicit signal and noise model. Second, in the more practical spectroscopic sense, resolution describes the ability of a method to detect and discriminate closely spaced peaks in a discrete spectral representation, under conditions of overlap and noise. The former notion can be quantified using theoretical limits such as the Cramér-Rao lower bound (CRLB) (*31*, *32*) and Bayesian Monte Carlo (MC) uncertainty estimates (*33*), whereas the latter is related to traditional resolution-enhancement procedures (*11*, *12*) and more recent DL-based algorithms (*29*). The ultimate resolution is understood in this work as a maximal achievable resolution limited by the information in the time domain signal and also constrained by practical discretizations of the spectrum. It is practically realized in a spectrum representation, where each peak is rendered with the minimal number of points and easily identified as a local maximum with its position accuracy closely approaching the theoretical limit.

Our approach is to define the problem in a precise statistical framework as determining the probability of finding the center of a signal at any given point in the spectrum. This definition shifts the focus to statistical analysis using DL (*34*).

The Bayesian approach has previously been used to define posterior probability distributions of spectral parameters in one-dimensional (1D) metabolomic spectra, leveraging a templated library of expected underlying compounds (*35*, *36*). However, applying traditional Bayesian methods to unconstrained multidimensional protein spectra is computationally prohibitive due to the extensive processing demands associated with Markov chain MC (MCMC) algorithms (*37*). DL circumvents this computational bottleneck by using the efficiency of deep learning in solving classification problems (*34*), enabling direct probability predictions. Reformulating the resolution problem as a classification task, peak or no peak, bridges the gap between statistical analysis and practical applications in multidimensional NMR.

NMR spectra are commonly analyzed in the frequency domain using traditional intensity presentation (TIP). Although this presentation is highly informative, TIP has notable drawbacks, including peak overlap exacerbated by the high dynamic range of signal intensities and challenges in distinguishing genuine peaks from spectral artifacts, which are often associated with high-amplitude signals, manifesting as pronounced line shape distortions, t1 noise, and NUS-related residual aliasing artifacts (*38*). As a complementary alternative to TIP, we introduce peak probability presentation (*P*^3^), a statistical representation applicable to NMR spectra of any dimensionality. *P*^3^ assigns, to each point in the spectrum, the probability of it being a peak maximum. This approach offers several advantages, including super-resolution, high sensitivity, a substantially reduced dynamic range, and effective suppression of spectral artifacts. To the best of our knowledge, *P*^3^ is the first statistical, nonparametric representation of a multidimensional spectrum. While its properties lie somewhere between peak-narrowing methods and peak-picking routines, *P*^3^ does not belong to either category.

We demonstrate that *P*^3^ provides a rectified spectral representation with near-ultimate resolution, supported by the information available in 2D and 3D spectra and by prior spectral knowledge embedded in the DL training set. *P*^3^ is computed using the newly developed magnetic resonance processing with AI (MR-AI) system, which can handle both conventionally acquired and NUS spectra. We demonstrate the approach using simulated spectra and, for real-world validation, curated datasets from the 100-Protein NMR Spectra database (*39*). We also showcase 2D and 3D spectra acquired for five prototypical systems, including challenging cases: the globular MALT1 protein (45 kDa) and the intrinsically disordered protein Tau (46 kDa). Last, the approach can leverage information transfer between spectra, which substantially improves results, as we demonstrate by analyzing a backbone-assignment experiment set for calmodulin, measured using a targeted acquisition (TA) data collection scheme (*40*, *41*).

## RESULTS AND DISCUSSION

### The peak probability presentations, *P*^3^

For a spectrum represented on a discrete grid, resolution may be defined as the accuracy in determining the position of a peak maximum, quantified in terms of grid points. This accuracy is degraded by low signal-to-noise ratios (SNRs), peak broadening, spectral overlap, and interference from larger peaks. Furthermore, it can be shown (see fig. S1) that the DFT, commonly used to convert a digital time-domain signal into a digital frequency-domain spectrum, imposes a theoretical resolution limit of approximately two to three grid points. The sharpest spectral peaks arise from non-decaying time-domain signals. Before applying the DFT, such a signal must be zero-padded to at least twice its original length. Although this procedure does not change the underlying information content of the time-domain data, owing to the causality principle (*5*, *6*, *42*), zero padding causes an exchange of information between the absorption- and dispersion-mode spectra, thereby enhancing and equalizing the information content in each. As a result, one spectrum can be obtained from the other using the Kramers-Kronig (Hilbert transform) relations. This is essential for optimal sensitivity and improving the digital sampling of the spectrum. The discontinuity between the original signal and the zero-padded segment must then be smoothed using a suitable weighting function to suppress sinc-shaped oscillations in the resulting spectrum. The Fourier transform (FT) of the thus-processed time-domain signal produces spectral peaks that typically span two to three grid points, with additional broadening introduced if the time-domain signal decays.

The ultimate resolution enhancement beyond the intrinsic limits set by natural line width and the properties of the FT can be achieved through nonlinear methods guided by rigorous statistical frameworks. To solve this task, we designed a deep neural network (see Methods for more details), which is trained to convert the traditional intensity presentation of the phase-sensitive NMR spectrum into the *P*^3^ form. In the *P*^3^, the typical precision of defining the peak maxima is one to two spectral points, which surpasses the theoretically possible resolution achievable in the traditional DFT-based intensity presentation of the phase-sensitive NMR spectra.

A crucial aspect of deep neural network design is selecting an appropriate cost function, which must be closely aligned with the chosen output unit activation function. Both the cost function and activation function depend on the specific task. For instance, in regression problems such as NUS reconstruction, Echo reconstruction, or virtual decoupling, a rectified linear unit activation function (*43*) combined with mean square error loss function is commonly used (*19*). Conversely, to estimate uncertainties of the spectra intensities, the negative log-likelihood loss function is required (*30*). In the case of *P*^3^, the network solves a binary classification problem with two classes. For each point in the spectrum, the peak maxima points are labeled as 1, whereas all other points are labeled as 0 (y∈0,1). Here, the binary cross-entropy (BCE) loss function, combined with a sigmoid activation function, provides an effective solution for inferring the probability estimation from a given training data (*34*).

The sigmoid function maps outputs of the neural network model into probabilities in the range [0, 1]

$$\sigma \left(x\right)=\frac{1}{1+e^{-x}}$$ (1)

This property makes it ideal for calibration of binary classification tasks, where the output represents the likelihood of belonging to the peak maxima class. For a Bernoulli distribution, the probability of observing a label *y* given a predicted probability *p* is defined as

$$P\left(y|p\right)=p^{y}{\left(1-p\right)}^{\left(1-y\right)}$$ (2)

Taking the negative log of this likelihood yields the BCE loss function

$$\text{BCE}=-\frac{1}{N}\sum_{n=1}^{N}[y_{n}\text{log}\left(p_{n}\right)+\left(1-y_{n}\right)\text{log}\left(1-p_{n}\right)\text{]}$$ (3)

By minimizing BCE during the network training on *N* different training data, the model optimizes its outputs to assign probabilities, which are close to 1 for defined peak maxima and have well-calibrated values down to zero for all other cases. This approach corresponds to maximizing the likelihood under a Bernoulli distribution.

The quality of the trained model, defined as correctness of the predicted probability distribution, can be accessed using appropriate statistical scores (*44*), including the normalized values of BCE and the Brier score (BS) defined as

$$\text{BS}=\frac{1}{N}\sum_{n=1}^{N}{\left(y_{n}-p_{n}\right)}^{2}$$ (4)

The true peak maxima are few in comparison to the prevailing number of other points in a multidimensional spectrum. In consideration of this strong minority class bias, it is convenient to compare the BCE and BS score values with those provided by the reference model that always predicts the base rate of the event. In this case, π is defined as the observed event frequency, i.e., the minor class probability

$$\pi =\frac{1}{N}\sum_{n=1}^{N}y_{n}$$ (5)

Then, the reference model always outputs a constant probability equal to the empirical event rate π where corresponding reference scores are equal to ${\text{BS}}_{\text{ref}}=\pi \left(1-\pi \right)$ and ${\text{BCE}}_{\text{ref}}=-\left[\pi \text{log}\pi +\left(1-\pi \right)\text{log}\left(1-\pi \right)\right]$.

The normalized BS or BCE scores, also called skill scores BSS and BCE-SS, respectively, are defined as

$$\text{Skill}=1-\frac{S}{S_{\text{ref}}}$$ (6)

where *S* stands for the BS or BCE score. A skill score (SS) equal to 1 corresponds to a perfect binary predictor and values equal or higher than 0.5 indicate a very good model.

In addition, the recall (the rate of the correctly predicted values), precision (the ratio of correctly predicted to all predicted values), F1 score (a balance between the recall and precision) metrics (*45*), and the probability calibration curves also known as the reliability diagram (*46*) are needed to analyze quantitatively the model prediction and calibration.

### High-resolution and low dynamic range in *P*^3^

**Fig. 1.**
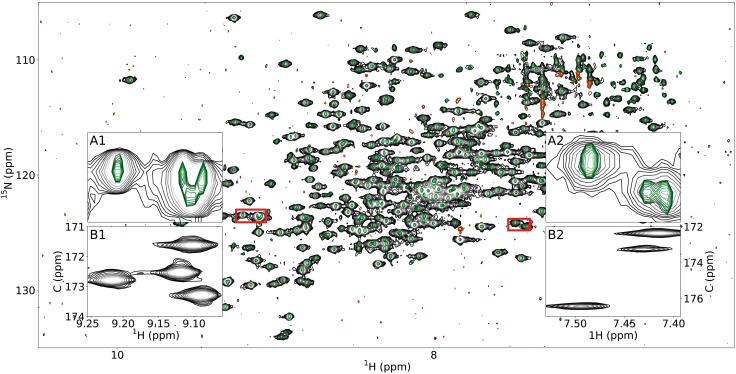
The *P*^3^ of experimental 2D NMR data. 2D ^1^H-^15^N–TROSY spectrum of MALT1 protein is shown with the black (positive) and orange (negative) contours, while the corresponding *P*^3^ spectrum is depicted in green. Inset panels *A*_1_ and *A*_2_ highlight zoomed-in regions (marked with red rectangles) of the spectrum; *B*_1_ and *B*_2_ confirm the resolved peaks in *A*_1_ and *A*_2_ by showing the corresponding ^1^H-^13^C slice from the 3D HNCO spectrum.

Figure 1 demonstrates the excellent performance of MR-Ai in generating *P*^3^ for the 2D ^1^H-^15^N correlation spectrum of MALT1 (45 kDa) (*47*, *48*) protein. Similar results for ubiquitin (7 kDa) (*49*), azurin (14 kDa) (*50*), and Tau (disordered, 45.8 kDa) (*51*) proteins are provided in figs. S2 to S4, respectively.

The *P*^3^ representation in Fig. 1 demonstrates significant line narrowing across the 2D spectrum while accurately reflecting both strong and weak peaks. The two insets in Fig. 1 confirm the improved resolution by displaying the peaks resolved in the 2D *P*^3^ alongside with the corresponding ^1^H-^13^C slice from the 3D HNCO spectrum. The *P*^3^ results for a set of 3D NUS experimental spectra used for the backbone assignment of MALT1 and calmodulin (17 kDa) (*52*) proteins are shown in figs. S5 to S15. Although these distortions were not present in the training set, MR-Ai appears capable of suppressing spectral features that do not resemble genuine peaks in the training set. Since no other significant spectral artifacts were observed in the used experimental spectra, further tests and possibly adjustment of the training dataset are necessary to generalize the method to other spectra cases and types, such as 2D and 3D nuclear Overhauser effect spectroscopy spectra.

**Fig. 2.**
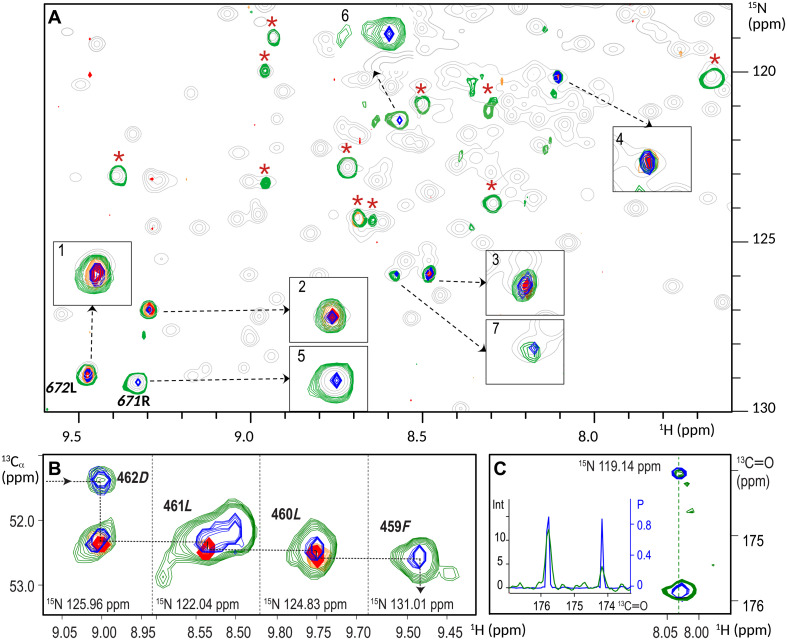
*P*^3^ disambiguates assignment in MALT1 spectra. (**A**) Superposition of the ^1^H-^15^N 2D planes, extracted at ^13^C^α^ frequency of 671*R*: 55.01 ppm from 3D HNCA (green and blue for TIP and *P*^3^) and HN(CO)CA (orange and red for TIP and *P*^3^) spectra and 2D TROSY spectrum (gray). Sequential (*i*) − (*i* − 1) peak labeled 672*L* and enlarged in inset box 1 has chemical shifts ^1^H*_i_*, ^15^N*_i_*, and ^13^C_*i*–1_ from residues 672*L* and 671*R*. The target (*i* − 1) cross-peak with chemical shifts ^1^H_*i*–1_, ^15^N_*i*–1_, and ^13^C_*i*–1_ of residue 671*R* is enlarged in box 5. Three additional sequential (*i*) − (*i* − 1) and two (*i* − 1) peaks appear in the plane because they exhibit ^13^C chemical shifts similar to that of 671*R*. These peaks correspond to residues 679*K*-678*L* (peak 2), 457*Q*-456 *L* (peak 3), 534*E*-533*A* (peak 4), 533*A* (peak 6), and one unidentified peak (*7*). Compared with *P*^3^, due to limited ^13^C resolution, the 3D HNCA (green) and HN(CO)CA (orange) planes also include eleven additional cross-peaks, marked with red stars making the assignment procedure more complicated. (**B**) Superposition of four strips ^1^H-^13^C 2D planes, extracted at ^15^N: 125.96, 122.04, 124.83, and 131.01 ppm, respectively, from 3D HNCA (green and blue for TIP and *P*^3^) and HN(CO)CA (orange and red for TIP and *P*^3^) spectra: The dashed line illustrates the flow of assignments through (*n*) to (*n* − 1) cross peaks, starting with residue 462*D*, and continuing through 461*L*, 460*L*, and 459*F*. (**C**) Superposition of the ^1^H-^13^C 2D planes, extracted at ^15^N: 119.14 ppm from 3D HN(CA)CO (green and blue for TIP and *P*^3^) spectra: The inside panel displays 1D projections corresponding to the dash column at ^1^H: 8.038 ppm, representing the chemical shifts of residue 545*K* and 544*G* where the vertical axes (on the right and left) are scaled according to intensity (Int) and probability (P), respectively.

Figure 2 illustrates the application of *P*^3^ to the backbone assignment of the 45-kDa MALT1 protein using 3D HNCA and HN(CO)CA spectra. The assignment progresses from residue 672*L* to its (*i* − 1) preceding residue 671*R* through visual inspection of the ^1^H-^15^N 2D planes extracted from the two 3D spectra at ^13^C: 55.01 ppm, corresponding to the ^13^Cα frequency of (*i* − 1) 671*R*. In the crowded spectra of MALT1, this assignment is substantially complicated by the presence of multiple candidate cross-peaks for (*i* − 1) 671*R*. Figure 2A shows numerous cross-peaks in HNCA (green contours) and HN(CO)CA (orange contours), all of which must be carefully evaluated to establish the correct sequential connection. The ambiguity, arising from insufficient resolution in the ^13^C dimension of traditional spectra, is largely alleviated in the *P*^3^ representation (Fig. 2A, blue for HNCA and red for HN(CO)CA). The enhanced resolution eliminates most of the irrelevant peaks (marked by red stars). Among the remaining seven peaks, only peak 5 (the correct 671*R* peak) and peaks 6 and 7 are retained in the *P*^3^ of the HNCA spectrum.

Another example of the superior resolution along the ^1^H and ^13^C dimensions in the *P*^3^ representation of the MALT1 3D HNCA and HN(CO)CA spectra is shown in Fig. 2B. This figure illustrates the assignment walk from 462*D* to 459*F*, tracing (*i*) to (*i* − 1) cross-peaks in four ^1^H-^13^C 2D strips extracted at ^15^N: 125.96, 122.04, 124.83, and 131.01 parts per million (ppm), respectively. The assignment walk is particularly challenging when relying solely on traditional 3D HNCA (green contours) and HN(CO)CA (orange contours) spectra, often necessitating additional experiments. In contrast, the walk based on better resolved *P*^3^, shown in blue (HNCA) and red (HN(CO)CA), is highly reliable, offering improved confidence in peak identification.

A remarkable difference in signal dynamic range between the traditional intensity presentation and *P*^3^ is illustrated in Fig. 2C, which shows a 2D strip extracted from the 3D HN(CA)CO spectrum for residue 545*K* at ^15^N: 119.14 ppm. Two cross-peaks, observed at 174.07 and 175.87 ppm, correspond to the carbonyl ^13^C frequencies of 544*G* and 545*K*, respectively. A 1D slice taken through these two cross-peaks at ^1^H: 8.038 ppm reveals a significant difference in the relative peak amplitudes. In the *P*^3^ representation, both peaks exhibit high and nearly equal probability (about 0.8), whereas in the conventional spectrum, their amplitude ratio is 3:1. Notably, in 3D HN(CA)CO spectrum, optimized for detecting ^1^H-^15^N-^13^C(i) cross-peaks corresponding to a residue’s own carbonyl carbon, the second peak, which corresponds to the carbonyl of the preceding (*i* − 1) residue, typically has lower intensity or may even disappear. The ability to simultaneously visualize all reliably detected signals, regardless of their intensity, significantly simplifies manual spectral analysis. Apart from MALT1, we illustrate the *P*^3^ on the set of triple-resonance 2D and 3D NUS experiments typically used for protein backbone assignment. The results are shown in the Supplementary Materials for five proteins of different sizes and spectra complexity.

### Large-scale benchmarking across 60 proteins

To evaluate the generality and robustness of the *P*^3^ representation across a diverse set of biomolecular NMR systems, we conducted a large-scale benchmarking study using the 100-Protein NMR Spectra Database (*39*), which provides broad coverage of different protein sizes, folds, and spectral complexities. Since the dataset contains only fully sampled spectra, we generated NUS datasets by applying NUS schedules. This allowed a direct assessment of the performance of *P*^3^ in both fully sampled and NUS-reconstructed spectra.

For each protein, experiment type, and NUS level, the spectra were reconstructed using the Compressed Sensing Iterative Soft Thresholding (CS-IST) algorithm (*53*), followed by conversion to the *P*^3^ representation, where peak lists were produced using a simple *P*^3^-based peak picker—*P*^5^. For the same reconstructed spectra, we also produced peak lists using the ARTINA peak picker at the NMRtist platform (*54*, *55*). For the peak lists obtained at several NUS sampling levels (5 to 100%), we computed recall, precision, and F1 scores. As the ground truth for the scores calculation, we used peaks generated from the manually curated chemical shift assignments included in the database. In addition, the practical value of the peak lists for the subsequent analysis was assessed by comparing the backbone assignment automatically produced by Combined Assignment and Dynamics Algorithm for NMR Applications (CYANA) and Flexible and Automated NMR Resonance Assignment (FLYA) (*56*) with the corresponding reference assignments.

Figure 3 illustrates quality metrics of the peak lists for seven representative proteins from the 100-Protein database (complete results for all proteins and sampling levels are found in the figs. S16 to S27). For fully sampled spectra, the MR-Ai workflow achieves recall comparable to ARTINA but consistently yields higher precision and F1 scores. This validates high sensitivity of the *P*^3^ MR-Ai method and its ability to suppress noise and spurious baseline features in both fully sampled and NUS spectra.

**Fig. 3.**
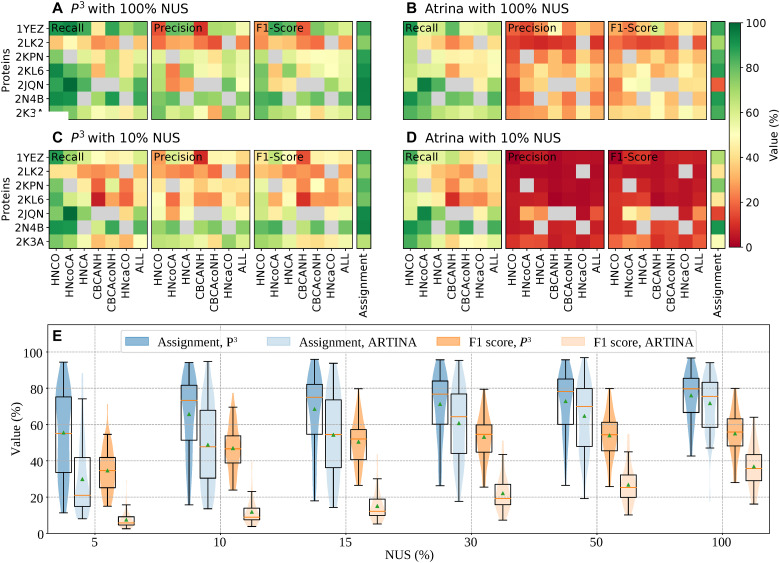
The quality scores for *P*^5^ peak lists obtained for seven representative proteins indicated by their four-letter Protein Data Bank (PDB) codes. Results for all 60 proteins and other NUS levels are found in figs. S16 to S21. (**A** and **C**) Results for MR-Ai. (**B** and **D**) Results for ARTINA. Panels (A) and (B) show the recall, precision, and F1 scores for fully sampled (100%) spectra. Panels (C) and (D) are the same metrics for 10% NUS data. For each protein (rows), the score values are color-coded for six individual triple-resonance backbone experiments [HNCO, HN(CO)CA, HNCA, CBCANH, CBCA(CO)NH, and HN(CA)CO], and ALL indicating combined scores across all experiments. An additional column shows the accuracy of the assignments. If, for a protein, a spectrum was missing in the database, then the corresponding square is shown in gray. (**E**) Statistics of *P*^5^ peak-list quality and assignment performance across 60 proteins for different NUS levels (5, 10, 15, 30, 50, and 100%). The violin and box plots show the distributions of peak-detection F1 scores (orange) and CYANA assignment accuracies (blue) obtained using *P*^5^ and ARTINA peak lists.

When the sampling density is reduced to 10% NUS, the difference between MR-Ai and ARTINA peak lists becomes even more pronounced. ARTINA, which is designed and validated only for the fully sampled spectra, shows a clear decline in precision and F1 score for nearly all protein experiment combinations, whereas *P*^5^ peak lists retain high precision and maintain a high recall level, resulting in significantly higher overall F1 scores and a higher level of the assignment.

The results shown in Fig. 3 (A to D) for individual proteins and experiment types corroborate with an overall statistical view on the F1 metrics and assignment levels, which were calculated more than 60 proteins and presented in Fig. 3E (individual experiment-level recall and precision results for all NUS levels are provided in the figs. S22 to S27). For fully sampled spectra, the distributions of the F1 scores and assignment accuracies are similar for ARTINA and *P*^5^, with slightly higher median precision for the *P*^5^. At low NUS levels (5 to 15%), ARTINA’s performance declines sharply, with spectra yielding very low or near-zero F1 scores and correspondingly reduced assignment levels. In contrast, *P*^5^ maintains stable and high F1 scores even at 10% NUS, indicating strong resilience to sampling sparsity.

Together, the results obtained for 60 proteins from the 100-Protein database demonstrate that the *P*^3^ representation generalizes effectively across proteins, experiment types, and sampling conditions. The method’s high sensitivity and high practical resolution are confirmed by the high quality of the peak lists obtained by a simple peak picking of local maxima in the *P*^3^ maps.

### The error analysis of the P^3^ using synthetic spectra

Despite the excellent *P*^3^ performance observed by careful visual inspection of the spectra of MALT1, ubiquitin, azurin, calmodulin, Tau proteins, and automated analysis more than 60 proteins from the 100-protein database, the quantitative validation of *P*^3^ using experimental data remains difficult due to the absence of ground-truth peak labeling. To address this limitation, we performed cross-validation of *P*^3^ using synthetic spectra that closely reproduce features of the signal and noise observed in experimental spectra.

**Fig. 4.**
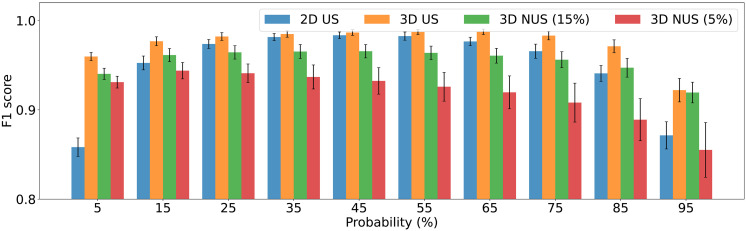
Statistics on peak detection of *P*^3^ in synthetic 2D and 3D Spectra. Blue, orange, green, and red bars with error bars represent the mean and SD of F1 score quality metrics across 20 synthetic NMR spectra, each containing 256 peaks, for US 2D, US 3D, 3D 15%, and 5% NUS reconstructed using CS-IST. F1 score is defined as the harmonic mean of the precision and recall scores with $F1=2\frac{\text{Precision}\times \text{Recall}}{\text{Precision}+\text{Recall}}$, where recall is defined as the ratio of the correctly detected pixels to all detectable pixels, while precision is the ratio of correctly detected pixels to all detected pixels. A pixel is considered as detected when its *P*^3^ value is above the probability threshold indicated on the horizontal axis of the chart. A pixel is correctly detected if it is found in the vicinity of a detectable pixel. The detectable pixels are defined as those near the maxima of the ground truth peaks with intensities higher than 2σ-noise for the US spectra. To account for the shorter experiment time in the 15% NUS spectra, a threshold of 5σ-noise from the corresponding US spectra was used.

Figure 4 presents the F1 score metrics (*45*) of *P*^3^, calculated across 20 2D and 20 3D synthetic spectra. Each spectrum contains 256 peaks with varying degrees of overlap and amplitudes, spanning a dynamic range of 1:200, with the weakest peak amplitudes down to one σ-noise. In all examined cases, including fully sampled 2D and 3D spectra as well as 3D NUS spectra, a favorable balance is observed between the number of spectral points correctly and incorrectly assigned to peak maxima. Thus, around a shallow optimum at ~50% probability, most true peaks are accurately detected, while the false detection rate remains very low. The selection of the probability threshold in practical applications depends on the task. For example, a lower probability cutoff, down to 10% or even less, may be preferable for the visual spectra analysis and automated assignment, as accepting a small number of false positives may be preferable to missing a few important low-intensity peaks.

Although 2D spectra exhibit a significantly higher degree of peak overlap compared to 3D spectra, *P*^3^ demonstrates remarkably consistent performance in both cases. Furthermore, as shown in Figure 4, the quality of *P*^3^ for 3D NUS-reconstructed spectra, which feature strongly non-Gaussian baseline noise, is comparable to that of uniformly sampled (US) data.

**Fig. 5.**
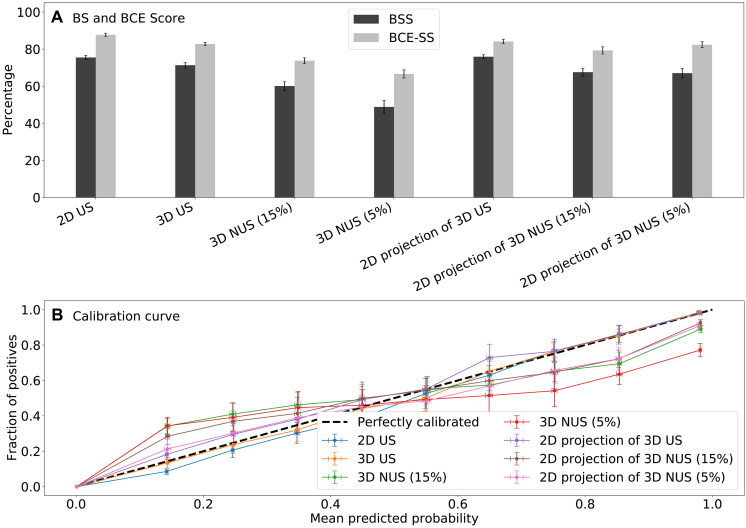
Statistics *P*^3^ calibration in Synthetic 2D and 3D Spectra. (**A**) BCE skill score (BCE-SS) and Brier skill score (BSS) (Eqs. 5 and 6) are calculated between predicted *P*^3^ and ground truth relative to baseline model across 20 synthetic NMR spectra, each containing 256 peaks, for 2D US, 2D projection of 3D US, 2D projection of 3D 15% and 5% NUS, 3D US, and 3D NUS reconstructed using CS-IST. The SS values equal or higher than 0.5 indicate a very good model. (**B**) Pixel-based calibration curves for *P*^3^ probabilities. Predicted probabilities are averaged within bins spanning 0.1 to 0.2, 0.2 to 0.3, etc. For each bin, the true fraction of the correct classifications is shown versus the averaged predicted probability. The ideal calibration curve is shown by the diagonal dashed line.

The probability estimates are considered well calibrated when the BCE and BS are significantly higher than the values for the baseline model. Figure 5A shows that the corresponding BCE-SS and BSS values are above 0.5, indicating excellent probability estimates.

In addition, Figure 5B and fig. S28 show the pixel-based and peak-based calibration curves mapping the predicted probabilities and actually observed classification rates. For all synthetic 2D and 3D spectra, the calibration curves are close to the ideal calibration (dashed line), illustrating the reliability of the *P*^3^ predictions for synthetic spectra.

**Fig. 6.**
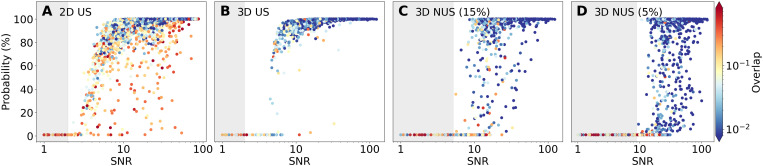
Effect of peak intensity and overlap on the peak detection in the *P*^3^ for the synthetic 2D and 3D Spectra. The probability of detecting peak maxima across 5120 ground truth peaks in 20 synthetic spectra are plotted versus the SNR and overlap conditions (color scale) for (**A**) 2D US, (**B**) 3D US, (**C**) 3D 15%, and (**D**) 5% NUS reconstructed using CS-IST spectra. For each ground truth peak with intensity *I*_0_, SNR is calculated as *I*_0_ divided by σ-noise in the US spectra (or the corresponding US spectra for NUS data). The overlap score is defined as $\Sigma_{i}\frac{I_{i}}{I_{0}d^{2}}$, where *I_i_* is the intensity of a neighboring peak *i* residing at a distance *d* less than 16 pixels. The gray areas indicate a range of signal amplitudes where the ground-truth peaks are theoretically undetectable with intensities below 2σ in the US spectra; to account for the shortened measurement time in the 15% and 5% NUS spectra, the theoretical detection threshold was set to 5 and 9, respectively, in units of σ-noise in the US spectra shown in (B).

Figure 6 illustrates the range of probability values assigned by MR-Ai to the positions of the ground truth peaks in the synthetic 2D and 3D spectra. The *P*^3^ values are influenced by the SNR of the peaks and the degree of overlap with the other peak. The latter is determined by the number of neighboring peaks, the distances to them, and their amplitudes compared to the peak in question.

For the 2D spectra (Fig. 6A), peaks with SNR < 3 or 4 have nearly zero probability values and are therefore not detected. This lower boundary for peak detection is close to the theoretical detection limit at a 5% confidence level (SNR = 2) and can also be attributed to the substantially higher degree of interference and overlap with other peaks, particularly for low-intensity peaks, as indicated by the color scale in Fig. 6. Although most peaks with SNR > 5 are detected with high confidence, a few medium-intensity peaks exhibit reduced probability values due to significant overlap. These peaks are located at the bottom of the charts. A similar pattern, although with less pronounced overlap, is observed in both 3D US spectra (Fig. 6B) and 3D 15 and 5% NUS spectra (Fig. 6, C and D). A notable feature of *P*^3^ in the 5% NUS 3D spectra is that the apparent peak detection threshold is very close to the theoretical limit defined by the noise level scaled to the reduced measurement time in NUS spectra. This highlights the near-ideal performance of both the CS-IST NUS spectrum reconstruction algorithm and the *P*^3^ produced by MR-Ai for synthetic spectra. The reduced probability values some peaks for the 5% NUS spectra (Fig. 6D) likely indicate both the sampling limit for CS-IST and border of the training domain for MR-Ai.

The results obtained from experimental spectra of several representative proteins, supported by quantitative validation using synthetic data, establish *P*^3^ as a powerful tool for significantly enhancing spectral resolution and simplifying analysis. Furthermore, *P*^3^ highlights new opportunities in NMR signal processing and analysis enabled by DL-driven approaches.

### Peak localization precision and resolution limits

To benchmark the quality of *P*^3^ maps produced by MR-Ai trained for the 2D ^1^H-^15^N correlation spectra, we compared precision of the peak positions obtained from the *P*^5^ peak picker with the fundamental statistical limits defined by the information contained in the time domain signal. We compared the *P*^5^ localization accuracy with three statistical error boundaries: the CRLB (*31*, *32*) and the precision provided by the Bayesian MCMC estimation (*33*) using either a Monte Carlo Weak prior (MCW) on the signal phase or a Monte Carlo Strong phase prior (MCS). The latter corresponds to the range of phase parameter values (see Methods), used both in the MR-Ai training and in generation of the spectra for calculating the statistics. The comparisons were carried out for 2D datasets containing (i) a single peak at different SNR and (ii) two peaks with varying degrees of overlap (see Methods for details) at fixed SNR = 10. The CRLB provides a theoretical lower bound on the variance of unbiased frequency estimates, while MCW and MCS represent practical Bayesian model parameter estimators with different levels of prior phase information.

**Fig. 7.**
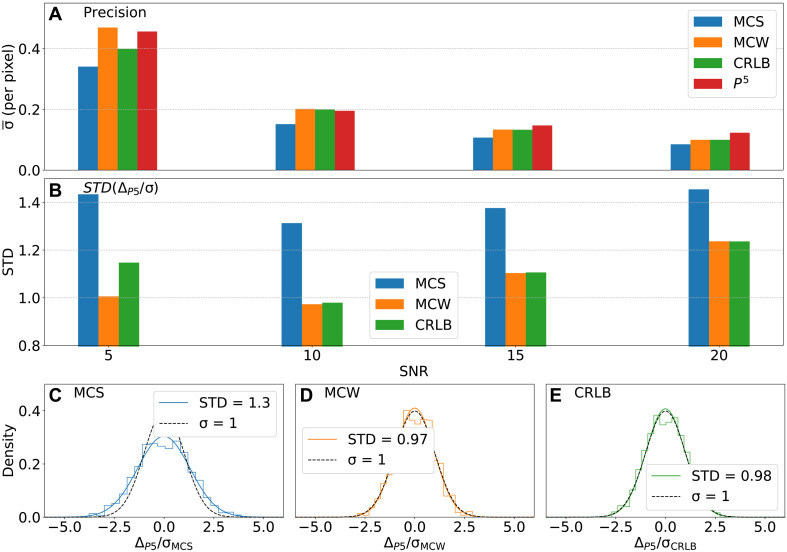
Comparison of *P*^5^ peak-list accuracy with CRLBs and Bayesian MCMC estimates. One thousand synthetic 2D spectra containing a single peak were generated with random frequencies, relaxation times, and phases (up to ±5°) at SNR values of 5, 10, 15, and 20. For each generated data, the frequency precision was estimated using three statistical approaches—CRLB (green), MCW with a weak phase prior (orange), and MCS with a strong phase prior (blue)—and compared to σ_*P*5_ = STD(Δ_*P*5_) (red), which is an SD of the *P*^5^ peak positions errors. (**A**) Mean localization precision $\bar{\sigma }$ (in spectrum pixels) as a function of SNR for all four methods. (**B**) SD of the normalized error Δ_*P*5_/σ for different σ values obtained from MCS, MCW, and CRLB across SNRs. Panels (**C** to **E**) show the distributions of Δ_*P*5_/σ at SNR = 10 for MCS, MCW, and CRLB, respectively, compared to a unit-variance Gaussian =1 (dashed). *P*^5^ achieves precision slightly better than MCW and CRLB while remaining less optimistic than MCS.

In the scenario of a single-peak at high SNR (SNR ≥ 15), the average localization precisions of CRLB and MCW converge because they use essentially the same signal models. MCS yields the smallest formal uncertainty due to its additional prior information about the signal phase (Fig. 7A). At SNR = 5 and 10, the precision of *P*^5^ approaches the CRLB/MCW limit and remains only slightly worse than the MCS case. At very low SNR (SNR = 5), however, the CRLB substantially underestimates the true variability, while MCW and MCS provide more realistic error estimates.

Figure 7B shows standard deviations of the *P*^5^ peak position errors normalized by CRLB, MCW, and MCS uncertainties. The values close to one for the CRLB and MCW normalizations indicate near theoretical precision for the *P*^5^ peak position, whereas higher than one values obtained when normalizing by the overconfident MCS uncertainties suggest about 40% lower than optimum precision. Similarly, plots of the distributions of the *P*^5^ normalized errors at SNR = 10 (Fig. 7, C to E) show that *P*^5^ achieves slightly better precision than MCW and CRLB but does not reach the optimistic MCS limit. These results indicate that *P*^5^ provides near-optimal and robust localization for isolated peaks, in the practically important low SNR ≤ 15 regime. For higher SNR, the theoretical limits continue to improve and fall below 0.1 pixel size, while *P*^5^ is limited by the digital resolution in the spectra.

**Fig. 8.**
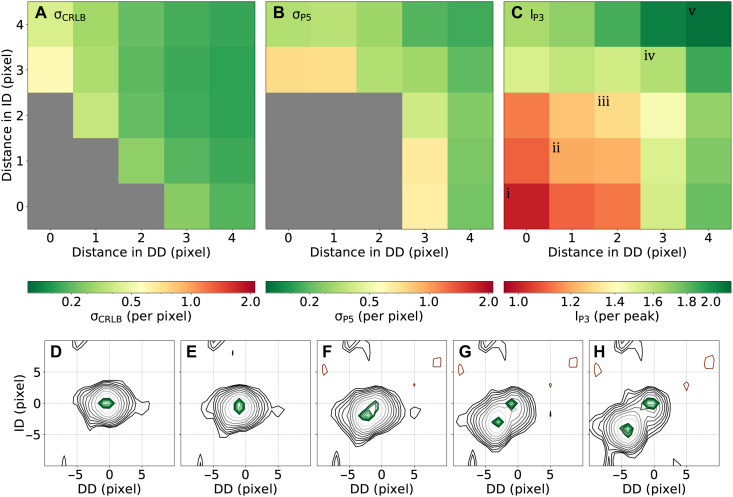
Two-peak resolution and relation between CRLB and *P*^3^/*P*^5^. One thousand synthetic 2D spectra were generated with a peak at SNR = 10, for which we access the position precision, and a second peak of twice the intensity placed at different distances in the direct (DD) and indirect (ID) dimensions. The top row shows heatmaps of (**A**) the CRLB-based frequency uncertainty σ_CRLB_, (**B**) *P*^3^/*P*^5^ localization uncertainty σ_*P*5_ and (**C**) the integrated probability *I*_*P*3_ as a function of interpeak separation (in pixels). Gray regions in (A) indicate conditions where 3σ_CRLB_ exceeds the interpeak distance, rendering the separation theoretically impossible, and in (B) mark cases where *P*^5^ finds a single peak instead of two. The bottom row (**D**) to (**H**) shows spectra (black) and *P*^3^ (green) contour maps for five representative interpeak separations *i*-*v* indicated in (C).

Figure 8 illustrates the performance of *P*^3^/*P*^5^ in the spectra containing two peaks at varying degrees of overlap. In Fig. 8 (A and B), the area of strong overlap is marked in gray, where two peaks are not formally resolved. It is defined by either a lack of two distinct maxima in the *P*^3^ map or when separation between the two peaks is less than 3σ_CRLB_. The blind spot is smaller for the CRLB. This may be partly explained by considering that the CRLB is calculated for the two-peak model, while the *P*^3^ does not use prior knowledge about the number of peaks. Beyond the unresolved area, the observed localization precision σ_*P*5_ closely follows σ_CRLB_ in the formally resolvable regime (Fig. 8, A and B), demonstrating that *P*^5^ peak lists maintain near-CRLB precision for the ground-truth peaks.

It is worth noting that the integral of *P*^3^ probability, *I*_*P*3_, is a useful proxy for the number of peaks in a spectrum. This is reminiscent of traditional 1D NMR, where the integral of the spectrum is proportional to the number of spins in the sample. Figure 8C, depicting *I*_*P*3_ as a function of the peaks’ overlap, captures the transition from one to two peaks even when the peaks’ maxima are not resolved. The integral is close to one—consistent with a single peak at very small separations (Fig. 8, D and E), increases toward a value indicative of two peaks at the margin (Fig. 8F), and approaches value of two at larger distances, where two local maxima are observed (Fig. 8, G and H). In the latter cases, the accuracy of the *P*^5^ peak positions from the ground truth is close to σ_CRLB_.

Together, the analysis of the synthetic spectra shows that for isolated peaks, *P*^5^ achieves localization precision close to the Cramér-Rao bound and to MC estimates with realistic priors. For peak doublets, the precision approaches the theoretical Cramér-Rao resolution limit set by the time-domain signal, with noticeable deviations mainly in the most challenging regime near the separability boundary, where the *P*^3^ probability maps still carry information about the presence of two peaks, although two distinct local maxima are not observed. By benchmarking *P*^5^ peak lists against the CRLB and Bayesian statistical standards under controlled synthetic conditions, we assess how closely the *P*^3^/*P*^5^ framework approaches the fundamental limits of peak localization and resolution.

### Targeted acquisition with hyperdimensional QSP

TA is a NUS-based incremental data acquisition strategy, in which the spectral quality is assessed concurrently with the experiment, typically relative to a task-specific target, such as detecting a sufficient number of peaks for the assignment of protein backbone (*57*). TA provides essential feedback on experimental progress and enables optimization of data collection, thereby significantly reducing measurement time for lengthy experiments. In our original implementation of the TA for the protein backbone assignment, peaks in 3D NUS triple resonance experiments were detected at each TA step using a hyperdimensional extension of the multidimensional decomposition (MDD) (*41*, *58*). This method leverages the significant sensitivity enhancement and robustness offered by the spectra coprocessing, the approach that is often used in the reconstruction of NUS spectra (*59*), real-time spectroscopy (*60*, *61*), and quantitative analysis of relaxation data (*62*, *63*).

However, generalizing MDD for coprocessing various combinations of spectral types and analysis tasks remains a challenge. In this work, we present the first proof-of-concept demonstration of a new general approach based on DL and statistical analysis for the spectra coprocessing and hyperdimensional spectroscopy (*58*, *64*).

**Fig. 9.**
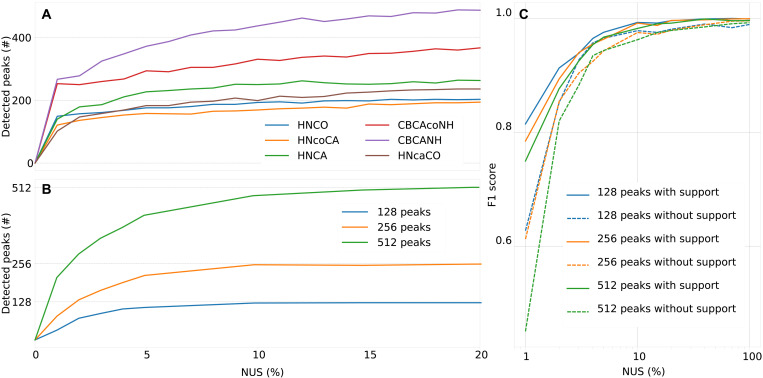
TA with the *P*^3^-derived peak lists. The number of detectable peaks estimated with the *P*^3^-based peak picker *P*^5^ (see Methods) in different 3D spectra reconstructed using CS-IST is plotted versus NUS fraction increasing during in course of the TA acquisition. (**A**) *P*^5^ peak counts in (at 20% probability cutoff) six color-coded experimental 3D spectra of calmodulin protein (16.7 kDa). The *P*^3^ was calculated using the 2D ^1^H-^15^N projection of the most sensitive 3D 50% NUS HNCO spectrum as a support. (**B**) *P*^5^ peak counts in synthetic 3D NUS HNCO-type color-coded spectra with 128, 256, and 512 peaks (blue, orange, and green, respectively) and the *P*^3^ were reconstructed using hyperdimensional coprocessing with the corresponding supporting 2D spectrum. (**C**) Enhancement of the peak detection in *P*^3^ of synthetic 3D spectra by their coprocessing with a supporting 2D spectrum. F1 score, which reflects a balance between the recall and precision, is defined as the harmonic mean of the precision and recall scores with $F1=2\frac{\text{Precision}\;\times \;\text{Recall}}{\text{Precision}\;+\;\text{Recall}}$. Recall is defined as the ratio of the correctly detected pixels to all detectable pixels, while precision is the ratio of correctly detected pixels to all detected pixels. A pixel is considered as detected when its *P*^3^ value is above the probability threshold of 40%. A pixel is correctly detected if it is found in the vicinity of a detectable pixel. The detectable pixels are defined as those near the maxima of the ground truth peaks with intensities higher than $2\sigma_{\text{noise}}/\sqrt{{\text{NUS}}_{\text{rate}}}$ in different NUS percentages indicated on the horizontal axis of the chart.

In Fig. 9A, the hyperdimensional coprocessing in MR-Ai (see Methods) is applied to a set of backbone assignment experiments acquired for calmodulin protein (16.7 kDa). The curves show a typical TA build-up of peaks counts, where the number of detected peaks increases as more NUS data are collected, with little further improvement beyond ~10% NUS, as confirmed by visual inspection of the spectra. Notably, the plateau values of the curves correspond closely to the expected number of peaks for each experiment type in the protein. The TA buildup curves of peak numbers in individual 3D spectra of calmodulin closely resemble those obtained using the original TA procedure (*41*) on the same spectra. These peak counts were obtained at a conservative 20% probability cutoff needed for the robust real-time acquisition monitoring. At the fine analysis stage, one may use a less strict cutoff allowing higher recall in expense of reduced precision. Figure 9B illustrates further validation of the TA approach using synthetic 3D spectra reconstructed with CS-IST across a broad range of NUS rates. Increasing the number of peaks in the spectra from 128 to 512 results in the corresponding scaling of the *P*^5^ peak numbers, confirming the method’s linear response to the number of detectable true peaks.

Figure 9C highlights the advantage of hyperdimensional coprocessing across all NUS point fractions. The improvement is especially pronounced at low NUS fractions, where coprocessing with a supporting 2D spectrum maintains a reliable F1 score, while unsupported analysis often yields poor performance. At low NUS levels, the limited acquisition time provides insufficient information for MR-Ai. The coprocessing introduces additional information from the support data and enables more accurate predictions and detection of more peaks in a broad range of NUS levels. This enables the real-time assessment of spectrum quality starting from the early stages of the TA process.

In this work, we leverage the power of DL to pave the way toward the ultimate resolution attainable through signal processing of multidimensional NMR spectra. We introduce *P*^3^, a new type of statistical spectral representation designed to enhance resolution while suppressing noise and spectral artifacts. The *P*^3^ is generated by a newly presented, computationally efficient MR-Ai architecture based on a physics-aware, cross-objective framework, generalized for arbitrary dimensionality. The performance of *P*^3^ is validated by extensive statistical analysis of synthetic 2D and 3D NUS spectra. Its practical utility is demonstrated through the analysis of 3D NUS spectra of 60 proteins from the 100-Protein NMR Spectra Database (*39*) and exemplified on several prototypical protein systems, encompassing a wide range of spectral complexity—from small globular proteins such as ubiquitin (8.6 kDa), azurin (14 kDa), and calmodulin (17 kDa) to larger and more challenging systems such as the globular MALT1 (45 kDa) and the intrinsically disordered Tau (45.8 kDa). Furthermore, we demonstrate a proof-of-principle application of MR-Ai for spectral coprocessing, as illustrated by hyperdimensional spectral analysis and TA. The results demonstrate power of the DL in the statistical analysis of complex NMR spectra.

## METHODS

### MR-Ai architecture for nD pattern

We introduce a generalized version of our magnetic resonance processing with AI (MR-Ai) architecture, designed to handle NMR spectra of any dimensionality. The MR-Ai framework was originally developed (*30*) for capturing 2D spectral patterns, including phase-twisted peaks associated with P-type (or N-type) data in the frequency domain (*30*). The original MR-Ai was, in turn, based on our earlier deep neural network architecture, WaveNet-based neural network (WNN) (*19*), which was designed to analyze 1D NMR frequency-domain spectra. The WNN model effectively captures defined spectral features, such as specific patterns of NUS aliasing artifacts and peak multiplicities in homonuclear decoupling experiments.

**Fig. 10.**
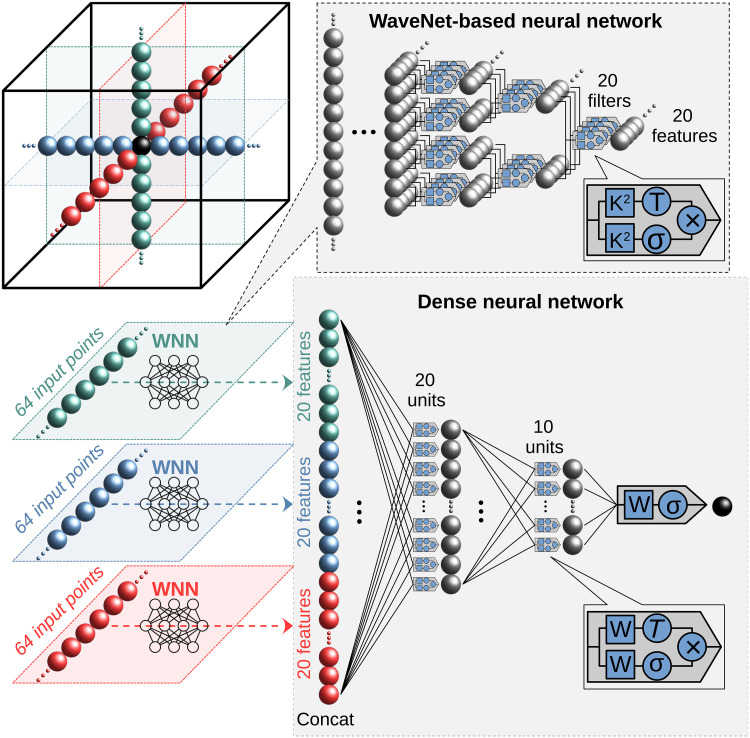
Schematic of MR-Ai architecture for 3D. Green, blue, and red points represent 64-point 1D cross sections along the three spectral dimensions. Each of these vectors, containing the examined point in the middle shown in black, is processed by an individual WNN, resulting in an output feature vector with 20 elements. The outputs are subsequently concatenated and fed into a DNN for further processing. The WNN architecture within MR-Ai extracts 20 features based on the number of 20 convolution filters applied. Symbols ×, *T*, and σ indicate element-wise multiplication operations, hyperbolic tangent activation, and sigmoid activation functions, respectively. The *K*^2^ and *W* denote learnable filter kernels of size 2 in 1D convolutional layers of the WNN and weights used in the DNN, respectively.

The new MR-Ai, referred to in this paper simply as MR-Ai, captures specific spectral patterns along the multidimensional cross in Cartesian coordinates, centered at a probed spectral point (Fig. 10). This cross-objective representation of the nD spectrum is inspired by the physical model of the NMR signal as a tensor product of 1D shapes (*65*). While notably compact, the cross-field of view retains most essential signal features, including line shape, phase distortions, *t*_1_ noise, *J*-coupling multiplets, wiggles from spectral truncation, and NUS-related point spread function patterns. Using a larger field of view, for example, an nD box, would require more DNN parameters and, in most cases, introduce more noise than new information.

Figure 10 depicts the architecture of MR-Ai, designed for generating *P*^3^. The network hyperparameters were optimized to achieve highly accurate results with a minimal number of learning parameters (about 8000), thereby reducing the required training set size and speeding up the computation. Each vector within the cross-objective is first processed individually by the WNN module, which converts it into a 20-element feature vector. The WNN architecture consists of 1D convolutional layers with a stride of 2, effectively skipping one data point between each convolution operation. Each layer uses a kernel size of 2 (*K*^2^) and contains 10 filters using a combination of hyperbolic tangent activation functions (*T*) and sigmoid activation functions (σ), without padding between layers. The feature vectors generated by the final WNN layers for all spectral dimensions are concatenated and passed as input into a dense neural network (DNN) with two hidden layers, containing 20 and 10 units, respectively. Each hidden layer applies the same combination of hyperbolic tangent (*T*) and sigmoid (σ) activation functions. The output layer of the DNN consists of a single unit with a sigmoid activation function and uses BCE as the loss function and the stochastic ADAM optimizer (*66*) with the default parameters. This layer processes the concatenated feature vectors to generate the final output, representing the adjusted probability value for the central point within the cross-objective, informed by the surrounding spectral context.

### Training MR-Ai model for *P*^3^

The first challenge in training a deep neural network is acquiring a sufficiently large and diverse dataset. To effectively train the model, it is necessary to generate a substantial number of cross-objectives along with their corresponding labels (1 for peak maxima and 0 otherwise). This requires access to a large number of spectra with accurately annotated peak positions. Our previous studies demonstrated that synthetic data can serve as an effective proxy for realistic experimental NMR spectra (*19*, *30*). In this work, we train MR-Ai using synthetic spectra, generating 2^24^ (≈ 16 million) cross-objectives and their associated labels. While most previous efforts have primarily focused on signal modeling, we incorporate synthetic noise to better simulate spectra representative of real experimental conditions. We found that accurately modeling noise with an appropriate statistical distribution, as described below, is just as critical as modeling the signal itself, especially for NUS-reconstructed data, where low-intensity peaks are particularly affected.

#### Synthetic nD spectra with associated labeling

For training, nD NMR time domain a hypercomplex signal $X_{\text{FID}}\in {ℍ}_{\mathrm{nD}}$, usually called free induction decay (FID), can be presented as a superposition of a small number of exponential functions

$$X_{\text{FID}}\left(t_{1},\ldots ,t_{n}\right)=\sum_{j}A_{j}\prod_{n}e^{-t_{n}/\tau_{n_{j}}}e^{\pm i\left(2{\pi \omega }_{n_{j}}t_{n}+\phi_{n_{j}}\right)}$$ (7)

*J* and *N* run over the number of exponentials and dimensions respectively, where the *j*th exponential in *n*th dimensional has the amplitude *A_j_*, phase $\phi_{n_{j}}$, relaxation time $\tau_{n_{j}}$, and frequency $\omega_{n_{j}}$. The evolution time *t_n_* is given by the series 0, 1, ..., *T_n_* − 1, where *T_n_* is the number of complex points in *n*th dimension. The desired number of different FIDs for the training and testing set is simulated by randomly varying the above parameters in the ranges summarized in table S1 for 2D ^1^H-^15^N and 3D ^1^H-^15^N-^13^C correlation spectra.

We used Python libraries, including nmrglue (*67*) and NMRPipe (*68*), for reading, writing, and processing NMR spectra, as well as mddnmr (*65*). The US spectra **S**_nD_ were obtained with the standard nD processing steps, which included apodization, zero filling, FT, and phase correction.

A binary label matrix, matching the dimensions of each processed synthetic spectrum, was generated on the basis of the provided peak list. Matrix elements were assigned a value of one at indices corresponding to peak maxima and zero elsewhere. If a peak maximum fell between two data points, within a range of 0.25 to 0.75 units from each, then both adjacent elements were assigned a value of one.

#### Training model for 2D US spectra

To train the model for 2D US spectra, we generated 640 synthetic noise-free 2D US spectra with 256 positive peaks in each (using a 4:1 ratio for training and validation datasets) based on table S1. The noise was simulated by a random Gaussian-distributed signal in the time domain and subsequently processed to the frequency spectrum using the same procedure as for synthetic spectra. After normalization of the noise in the frequency domain, so that its SD matches to the smallest possible peak amplitude, the noise was added to the synthetic spectrum, **S**_nD_.

#### Training model for 3D spectra

To train the model for 3D spectra, we generated 1280 synthetic noise-free 3D US spectra with 256 positive peaks in each (using a 4:1 ratio for training and validation datasets) based on table S1. Groups of four spectra were added with positive and negative signs. This increased the number of peaks and overlaps and resulted in both positive and negative peaks. Similar to 2D US spectra, the 3D US spectra include Gaussian noise. However, we observed that in the case of 3D NUS spectra reconstructed using the CS-IST algorithm, the apparent baseline noise and artifacts do not follow a normal (Gaussian) distribution with gradually increased fraction of outliers as the number of NUS points decreases (see fig. S29A).

To simulate this behavior, we adopted a simple noise model that allows for outliers by combining Cauchy and Gaussian components. Their normalized relative contributions in the frequency domain were varied as a function of the NUS fraction, ranging from ~0:1 (purely Gaussian) for fully sampled data to 1:0 (purely Cauchy) for NUS levels below 5%. To this end, 5% NUS may be considered the border line for the training domain. Using Cauchy-featured noise instead of a purely Gaussian distribution during training enhanced the precision of the trained network without significantly compromising its sensitivity, as measured by the recall score (see fig. S30). After normalizing the synthetic noise in the frequency domain, it was added to **S**_nD_ as previously described.

### Resampling and the regions of interest

In classification tasks, an imbalance in class representation, where one class significantly outnumbers another, can lead to biased models that perform poorly on the minority class (*69*, *70*). In 2D spectra, where the class ratio (i.e., the number of points with and without spectral maxima) is ~1:100, this slight imbalance does not pose a significant problem. However, in 3D spectra, a severe imbalance arises due to the sparsity of 3D data, with class ratios exceeding 1:10^4^. Training the deep neural network on such an imbalanced dataset gives a model that is insensitive to low-intensity peaks. To mitigate this issue, resampling techniques, which adjust the training data to balance class distributions, can significantly improve model performance. Specifically, the minority class can be oversampled or the representation of the majority class cases in the training set can be reduced. (*69*, *70*). For training the model in the 3D case, we specifically undersampled data points associated with the background. This was achieved by selecting all points corresponding to peak maxima (labels with ones) and a subset of nonpeak points (labels with zeros), ensuring a class ratio of ~1:100. This approach not only improved the model’s sensitivity to small peaks but also led to an increase in false positive hits. This is expected, as even in an ideal 3D spectrum of typical size filled with Gaussian noise, more than 100 peaks are expected to exhibit intensities exceeding four SDs of the baseline noise. The number of noise-induced peak-like features is even greater in NUS-reconstructed spectra. To reduce the number of false positives, we focus the analysis on regions of interest, as described below.

#### Training model for 2D sky projections of 3D spectra

Although the trained network for 3D spectra can successfully detect even very low SNR peaks, it is also highly sensitive to spurious peak-like noise features and spectral artifacts in NUS spectra. Distinguishing real peaks from intense noise features and artifacts is nearly impossible without additional prior knowledge.

As it was noted above, the 2D spectra do not display a significant imbalance in the representation of the classes’ peaks versus nonpeaks. This allows direct detection of the low-intensity peaks. Moreover, even if the baseline noise in a 3D spectrum has a strong Cauchy-like deviation from Gaussian distribution, the noise in the 2D projections has a distribution featuring favorable properties of the normal distribution due to the central limit theorem (*71*). To leverage this property, we trained models to predict *P*^3^ values for all three 2D skyline projections of the 3D spectrum. The primary difference between 2D skyline projections of 3D spectra and conventional 2D US spectra lies in their noise distributions. While noise in 2D US spectra follows a Gaussian distribution, noise in 2D skyline projections from 3D spectra features a symmetric double maxima distribution. Figure S29B illustrates that across a broad range of NUS rates (15 to 100%), artifacts and noise in 2D skyline projections exhibit similar distributions, with slight variations in the middle gap and spread. To replicate this noise behavior during training, we used a simple bimodal (double Gaussian) distribution, in which the normalized separation between the two means was varied from 0 to 5 SDs. A separation of 0 (no gap) was used for the conventional 2D spectra, whereas the training with the separations from 1 to 5σ significantly improves recall by capturing the characteristics of 2D skyline projections from 3D spectra reconstructed under both US and different NUS rates (see also fig. S31).

### Production run of trained MR-Ai

For 2D US spectra, the trained MR-Ai model, designed to handle Gaussian-distributed noise, can be applied directly to predict *P*^3^. In the case of 3D spectra, *P*^3^ reconstruction is preceded by an intermediate step to identify regions of interest (ROI), as depicted in fig. S32.

First, *P*^3^ values are calculated for the three orthogonal 2D skyline projections using an MR-Ai model trained on 2D projections with noise modeled as a double Gaussian distribution. Then, each point in the original 3D spectrum is assigned a preliminary probability score, computed as the element-wise product of the corresponding probability values from the 2D *P*^3^ projections. Points in the 3D spectrum with the geometrical probability average over the used orthogonal projections exceeding 2.5% are selected as regions of interest. This represents a reasonable compromise between the highest recall in the 2Ds (Fig. 5 and figs. S30 and S31) and sufficiently narrow ROI to avoid class imbalance in processing the 3D spectra.

Last, spectral points within the regions of interest are evaluated using the sensitive 3D MR-Ai model, which was trained on noise modeled as a Cauchy-Gaussian distribution. It is worth noting that 2D projections can be directly measured in the time domain due to the Fourier Projection Theorem and have been successfully used in NMR for a long time, particularly in NUS spectrum reconstruction for multidimensional spectra (*72*–*74*).

MR-Ai enables the hyperdimensional analysis and coprocessing of multiple spectra, leveraging power of information transfer between the spectra (*58*, *59*, *64*). As shown in fig. S32, MR-Ai incorporates supporting spectra during the definition of regions of interest. For instance, for enhanced signal detection, an alternative high-sensitivity 2D spectrum (e.g., 2D HSQC) can be used instead of a 2D skyline projection from a processed 3D spectrum. As illustrated in Fig. 9C, this spectral support is particularly beneficial in the sampling-limited regime, where the number of available NUS points is too low for reliably resolving all the spectra signals.

### Computations

The MR-Ai network architecture and associated graphs were generated using the TensorFlow Python library (*75*) with the Keras front end (see Supplementary Text for Python code). The models were trained on the NMRbox server (*76*), equipped with 128 cores, 2 terabytes of memory, and 4 NVIDIA A100 Tensor Core GPUs. The learning rate of 0.001 and a mini-batch size of 2^16^ were empirically adjusted to optimize the convergence in the 2D and 3D cases. The trainings were terminated after 2^10^ epochs or earlier if the monitored validation metric stopped improving. Convergences of the training and cross-validation losses during the training of all MR-Ai models used for *P*^3^ prediction are shown in the fig. S33. Training the models typically required ~3 and 6 hours of GPU time for the 2D and 3D cases, respectively. In addition, the preparation of the synthetic training dataset took about one CPU hour for the 2D and 2 hours using 10 CPU cores running in parallel for the 3D. The data preparation time can be easily shortened by more extensive parallelization. Because of the lightweight network architecture consisting of ~8000 trained parameters, the production runs for standard 2D and 3D spectra do not require a GPU and take about 10 s and 1 min, respectively, on a standard MacBook Pro notebook equipped with a 2-GHz Quad-Core Intel Core i5 processor. No parameters need adjustment at the production stage.

### Synthetic test data

To perform error analysis of the trained MR-Ai model for the P3 and TA, we generated 2D and 3D US spectra based on table S1 and added Gaussian-distributed noise in the same way as for the training dataset. For the 3D NUS case, the 3D US spectrum was first down-sampled in the time domain to the desired number of NUS points. Subsequently, CS-IST was used for the spectrum reconstruction.

### Experimental test data

To test trained MR-Ai performances, we used previously described 2D and 3D spectra for several proteins: ubiquitin (*49*), azurin (*50*), Tau (IDP) (*51*), MALT1 (*47*, *48*), and calmodulin (*52*). The 2D US and 3D NUS experiments used in this study are described in table S2. We used NMRPipe (*68*), Python package nmrglue (*67*), mddnmr (*57*), and TopSpin (Bruker Biospin) software for reading, writing, and traditional processing of the NMR spectra. We used CS-IST (*53*) using the default mddnmr parameters and the Virtual-Echo mode (*42*) for 3D NUS reconstruction.

### Benchmarking with experimental spectra from 60 proteins

Large-scale statistical validation was performed using the 100-Protein NMR spectra dataset (*39*), which provides fully sampled 2D and 3D triple-resonance spectra together with manually curated resonance assignments. From this dataset, we selected 60 proteins for which at least four of the following 3D experiments were available: HNCO, HNCA, HN(CO)CA, HN(CA)CO, CBCANH, and CBCA(CO)NH. This subset ensures consistent representation of core experiment types used for protein backbone assignment across proteins with diverse sizes and spectral characteristics. Chemical shifts from the available reference assignments were used to generate expected peak lists for all experiment types. These peak lists were used as the ground truth for calculation of the peak quality scores.

Because the dataset contains only the fully processed frequency-domain spectra, these were first converted to the time domain using the Hilbert and inverse FTs, followed by undoing the zero filling. Then, the NUS data at multiple sampling densities were produced by applying the corresponding Poisson gap sampling schedules. All NUS data were zero-filled to four times the original size in all indirect dimensions, followed by the spectra reconstruction using the CS-IST algorithm implemented in MDDnmr software (*53*), with identical reconstruction parameters across all proteins and experiments.

For each protein and each NUS rate, the reconstructed spectra were submitted to the NMRtist platform (*54*) to obtain ARTINA peak lists and automated assignments. The same spectra were processed with MR-Ai to compute *P*^3^ maps, from which *P*^5^ peak lists were generated and subsequently used as input to CYANA/FLYA (*56*) for automated backbone assignment.

### Peak picker (*P*^5^) for *P^3^* maps

Peak lists were generated from the *P*^3^ representations using a dedicated probability-based peak picker, denoted as *P*^5^. For optimal peak quality, the input spectra were zero-filled twice before *P*^3^ generation. *P*^5^ identifies a candidate peak by locating local maximum in the *P*^3^ map and refining its coordinates using a three-point quadratic interpolation along each dimension. The inherent sharpness and low dynamic range of *P*^3^ render peak detection robust and precise, even in highly undersampled NUS spectra. The resulting peak lists contain both peak positions and associated probability values, which serve as confidence estimates for downstream analysis.

### CRLBs for 2D peaks

To quantify the theoretical limits of peak localization, each synthetic 2D signal $\mu \left(t_{x},t_{y}\right)$ was modeled as a sum of *J* exponentially decaying complex exponents sampled on a Cartesian grid Eq. 7 and corrupted by additive complex Gaussian noise $\varepsilon \left(t_{x},t_{y}\right)$ with variance σ^2^

$$\mu \left(t_{x},t_{y}\right)=X_{\text{FID}}\left(t_{x},t_{y}\right)+\varepsilon \left(t_{x},t_{y}\right)$$ (8)

where each peak is parameterized by an amplitude *A_j_*, relaxation times $\tau_{x_{j}}$ and $\tau_{y_{j}}$, frequencies $\omega_{x_{j}}$ and $\omega_{y_{j}}$, and phases $\phi_{x_{j}}$ and $\phi_{y_{j}}$ in the direct and indirect dimensions.

For a given ground-truth parameter set, all peak parameters were collected into a single vector

$$\theta ={\left(A_{1},\tau_{x_{1}},\tau_{y_{1}},\omega_{x_{1}},\omega_{y_{1}},\phi_{x_{1}},\phi_{y_{1}},\ldots ,A_{J},\tau_{x{,}_{J}},\tau_{y_{J}},\omega_{x_{J}},\omega_{y_{J}},\phi_{x_{J}},\phi_{y_{J}}\right)}^{⊤}$$ (9)

The Fisher information matrix F with respect to **θ** was then computed from the analytical derivatives of $\mu \left(t_{x},t_{y};\theta \right)$ in all four quadrature channels (real-real, real-imaginary, imaginary-real, imaginary-imaginary). Denoting by $\mu_{c}\left(t_{x},t_{y};\theta \right)$ the *c*-th quadrature component, the Fisher matrix elements are

$$F_{\mathrm{pq}}\left(\theta \right)=\frac{1}{\sigma^{2}}\sum_{t_{x}}\sum_{t_{y}}\sum_{c=1}^{4}\frac{\partial \mu_{c}\left(t_{x},t_{y};\theta \right)}{\partial \theta_{p}}\frac{\partial \mu_{c}\left(t_{x},t_{y};\theta \right)}{\partial \theta_{q}}$$ (10)

where the sums run over all sampled time points $\left(t_{x},t_{y}\right)$ and quadrature channels *c*.

The CRLB for each parameter θ*_p_* was obtained from the corresponding diagonal element of the inverse Fisher matrix

$$\text{Var}\left({\hat{\theta }}_{p}\right)\geq {\left[F^{-1}\left(\theta \right)\right]}_{\mathrm{pp}}$$ (11)

and we report the CRLB as the square root of this variance. Frequency uncertainties are reported in pixel units.

### Bayesian MC error estimates

To obtain MC estimates of peak-localization uncertainty, we constructed a Bayesian model of the same complex exponential signal as was used for the CRLB analysis. The observed data consisted of the real and imaginary parts of all four quadrature channels (real-real, real-imaginary, imaginary-real, and imaginary-imaginary). Weakly informative priors were assigned to the model parameters: half-normal distributions for amplitudes and relaxation times, normal priors for frequencies centered at their ground-truth values with widths corresponding to three times the spectrum digital resolution, and normal priors for phases. We considered a hierarchical Bayesian model with two phase priors: (i) a broad prior representing weak phase information (MCW, σ_ϕ_ = 30°) and (ii) a narrow prior representing strong phase information (MCS, σ_ϕ_ = 4°) related to the phase range in table S1. The noise SD σ was given a half-normal prior.

Posterior sampling was performed in the PyMC probabilistic programming library (*77*) using the NUTS algorithm with four chains, 2000 posterior draws, and 500 burn-in steps. For each realization and each SNR, the posterior SDs of the peak frequencies served as MC estimates of localization precision.

### Computation of *P*^3^ localization and resolution metrics

We evaluated statistics on the empirical precision and resolution of the *P*^3^ representation by testing it on two scenarios using 1000 2D synthetic spectra in each case.

#### Spectrum with a single-peak

For each realization, a single exponentially decaying complex peak with randomly drawn frequencies, relaxation times, and phases (up to ±5°) was generated and corrupted by complex Gaussian noise at SNR values of 5, 10, 15, and 20. For each spectrum, CRLBs and MC uncertainties (MCW and MCS) were computed as described in the previous subsections. The corresponding *P*^3^ maps were generated using the MR-Ai network. Peak positions were extracted using *P*^5^, and the root mean square error across the 1000 realizations provided the empirical *P*^3^ localization precision σ_*P*5_ for each SNR. To assess statistical calibration, for each peak, we computed the *P*^3^ localization error, Δ_*P*5_/σ normalized using σ obtained separately from the CRLB, MCW, and MCS estimates, and evaluated its SD across all realizations. This allowed us to test whether the nominal uncertainties from each estimator are consistent with the observed spread of *P*^3^/*P*^5^ localization errors.

#### Spectrum with two peaks

To assess resolution, at SNR = 10, we synthesized spectra containing two peaks—a reference peak and a second peak of twice the SNR placed at different separations in the direct and indirect dimensions. For each peak pair, we calculated the CRLB-based frequency uncertainty of the reference peak σ_CRLB_. Distances for which 3σ_CRLB_ exceeded the interpeak separation were classified as theoretically unresolvable from the time-domain signal. For each separation, we calculated the corresponding *P*^5^ peak localization precision σ_*P*5_ and the integrated probability *I*_*P*3_ over the entire two-peak cluster. The integral reflects the effective number of peaks in the cluster.
