# Supplementary material for: Toward ultimate NMR resolution with deep learning
Source: Sci Adv. 2026 Mar 27;12(13):eady7995. doi: 10.1126/sciadv.ady7995 (PMC13025101; doi:10.1126/sciadv.ady7995)
Supplement: Supplementary file 1 — Supplementary Text Tables S1 and S2 Figs. S1 to S33 [file sciadv.ady7995_sm.pdf]

Science Advances
AAAS

Supplementary Materials for

Toward ultimate NMR resolution with deep learning

Amir Jahangiri *et al.*

Corresponding author: Vladislav Orekhov, vladislav.orekhov@nmr.gu.se

Sci. Adv. **12**, eady7995 (2026)
DOI: 10.1126/sciadv.ady7995

This PDF file includes:

Supplementary Text
Tables S1 and S2
Figs. S1 to S33

1 Supplementary Methods

1.1 Supplementary Text: Python MR-Ai code for 2D and 3D P^3

```
import tensorflow as tf
import numpy as np

def WNN(layer, filter):
    def inner_function(x):
        for i in range(layer):
            t = tf.keras.layers.Conv1D(filter, kernel_size=2,
                                        strides=2, activation='tanh')(x)
            s = tf.keras.layers.Conv1D(filter, kernel_size=2,
                                        strides=2, activation='sigmoid')(x)
            x = t * s
        return x
    return inner_function

def DNN(filter):
    def inner_function(x):
        t = tf.keras.layers.Dense(2*filter,activation='tanh')(x)
        s = tf.keras.layers.Dense(2*filter,activation='sigmoid')(x)
        x = t * s
        t = tf.keras.layers.Dense(filter,activation='tanh')(x)
        s = tf.keras.layers.Dense(filter,activation='sigmoid')(x)
        x = t * s
        return x
    return inner_function

def WNN_2D(Size,filter,LR):
    I = np.log2(Size).astype(int)
    input = tf.keras.layers.Input(shape = [Size[0]+Size[1],1])
    x = tf.keras.activations.softsign(10*input)
    x_0, x_1 = tf.split(x, [Size[0],Size[1]], 1)
    x_0 = WNN(I[0],filter)(x_0)
    x_1 = WNN(I[1],filter)(x_1)
    x_0 = tf.keras.layers.Conv1D(3*filter, kernel_size = 1)(x_0)
    x_1 = tf.keras.layers.Conv1D(3*filter, kernel_size = 1)(x_1)
    output = tf.squeeze(tf.concat([x_0,x_1],-1), 1)
    output = DNN(filter)(output)
    output = tf.keras.layers.Dense(1,activation='sigmoid')(output)
    model = tf.keras.Model(inputs = input, outputs = output)
    model.compile(loss = tf.keras.losses.BinaryCrossentropy(),
                  optimizer = tf.keras.optimizers.Adam(learning_rate = LR))
    return model
```

```
def WNN_3D(Size,filter,LR):
    I = np.log2(Size).astype(int)
    input = tf.keras.layers.Input(shape = [Size[0]+Size[1]+Size[2],1])
    x = tf.keras.activations.softsign(10*input)
    x_0, x_1, x_2 = tf.split(x, [Size[0],Size[1],Size[2]], 1)
    x_0 = WNN(I[0],filter)(x_0)
    x_1 = WNN(I[1],filter)(x_1)
    x_2 = WNN(I[2],filter)(x_2)
    x_0 = tf.keras.layers.Conv1D(2*filter, kernel_size = 1)(x_0)
    x_1 = tf.keras.layers.Conv1D(2*filter, kernel_size = 1)(x_1)
    x_2 = tf.keras.layers.Conv1D(2*filter, kernel_size = 1)(x_2)
    output = tf.squeeze(tf.concat([x_0,x_1,x_2],-1), 1)
    output = DNN(filter)(output)
    output = tf.keras.layers.Dense(1,activation='sigmoid')(output)
    model = tf.keras.Model(inputs = input, outputs = output)
    model.compile(loss = tf.keras.losses.BinaryCrossentropy(),
                  optimizer = tf.keras.optimizers.Adam(learning_rate = LR))
    return model
```

2 Supplementary Tables

Table S1: **Parameters for the synthetic nD FID**

$A_n \in \mathbb{R}$	$\omega_{n_j} \in \mathbb{R}$	$\tau_{n_j} \in \mathbb{R}$ (DD)	$\tau_{n_j} \in \mathbb{R}$ (ID)	$\phi_{n_j} \in \mathbb{R}$	$T_n \in \mathbb{N}$
[0.05, 1]	[−0.5, 0.5]	[12.8, 64]	[64, 1280]	$\mathcal{N}(\mu = 0, \sigma = 1.5)$	128

Table S2: **Spectral parameters**

Protein	Ubiquitin	Azurin	Tau	MALT1	Calmodulin
Size	8.6 kDa	14 kDa	45.8 kDa	45 kDa	17 kDa
Concentration	0.6 mM	1 mM	0.5 mM	0.5 mM	1 mM
Spectrum	2D US (^{1}H, ^{15}N)				
	HSQC	HSQC	TROSY	TROSY	
				3D NUS (^{1}H, ^{13}C,^{15}N)	
				HNCO	HNCO
				HNCA	HNCA
				HN(CA)CO	HN(CA)CO
				HN(CO)CA	HN(CO)CA
				HNCACB	HNCACB
				—	HN(CO)CACB

3 Supplementary Figures

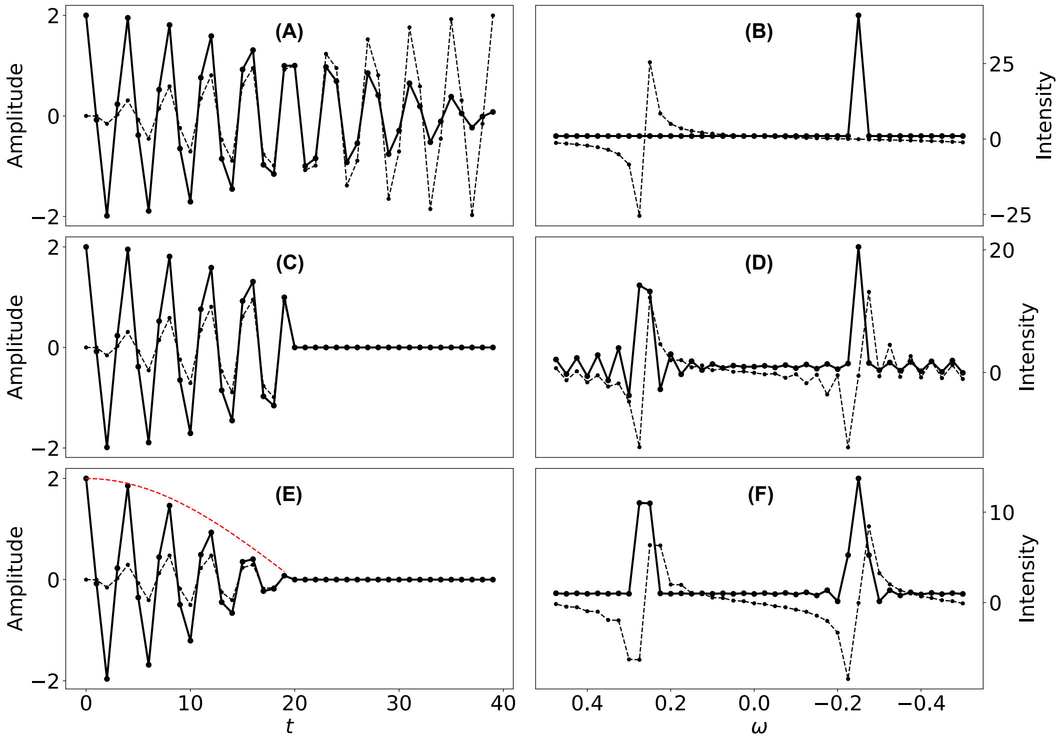

Figure S1: **Resolution limits in the spectra imposed by the discrete Fourier transform (DFT).** As can be easily shown by explicit DFT summations and illustrated in this figure, in the best-case scenario of a time signal including non-decaying and non-overlapping complex exponentials, the resolution is limited to 2-3 data points. The left and right panels show, respectively, discrete time-domain signals and the corresponding frequency spectrum obtained by the DFT. The real and imaginary parts in the complex time and frequency domains are depicted with solid and dashed lines, respectively. **(A)** a time domain signal including two non-decaying complex exponentials with the same amplitude and zero phase. **(B)** the spectrum obtained by DFT from **(A)**. Note that this spectrum cannot be phased because the left signal is presented only by the imaginary dispersion part, while the right peak has only the real absorption part. The two peaks are different only in their frequency positions, with the left peak residing in the middle between two points in the discrete spectrum, while the right peak sits exactly at one of the spectrum points. **(C)** Half of the signal from **(A)** is padded with zeroes to the original size. The corresponding spectrum **(D)** can be phased but exhibits sinc-oscillations, causing interference between the peaks. **(E)** Signal from **(C)** multiplied by the weighting function shown in red. **(F)** A traditionally looking spectrum of **(E)** which avoids problems of **(B)** and **(D)** with spectral peaks spanning 2-3 data points.

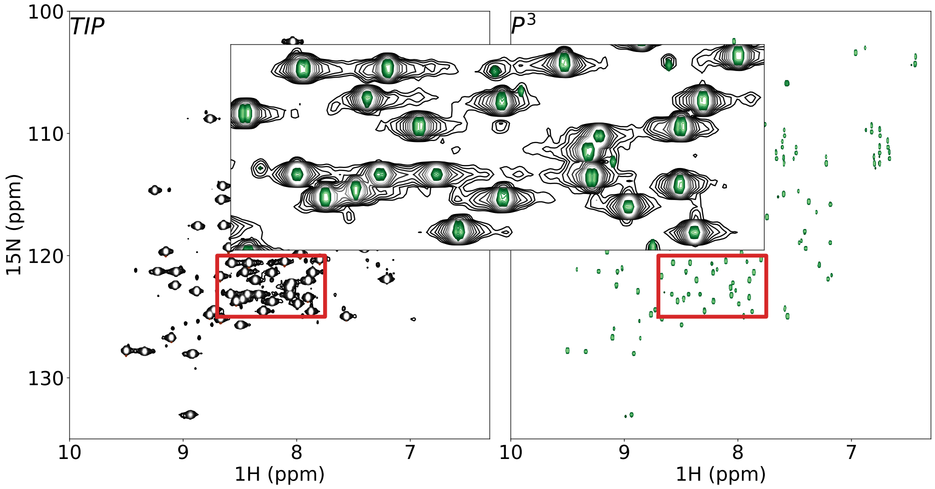

Figure S2: **2D ^{1}H-^{15}N — HSQC spectrum of Ubiquitin protein.** Traditional intensity presentation, TIP, in black and peak probability presentations, P^3 by using MR-Ai, in green color. Note that many homonuclear couplings are visible in the ^1H dimension due to high resolution in the P^3 presentation.

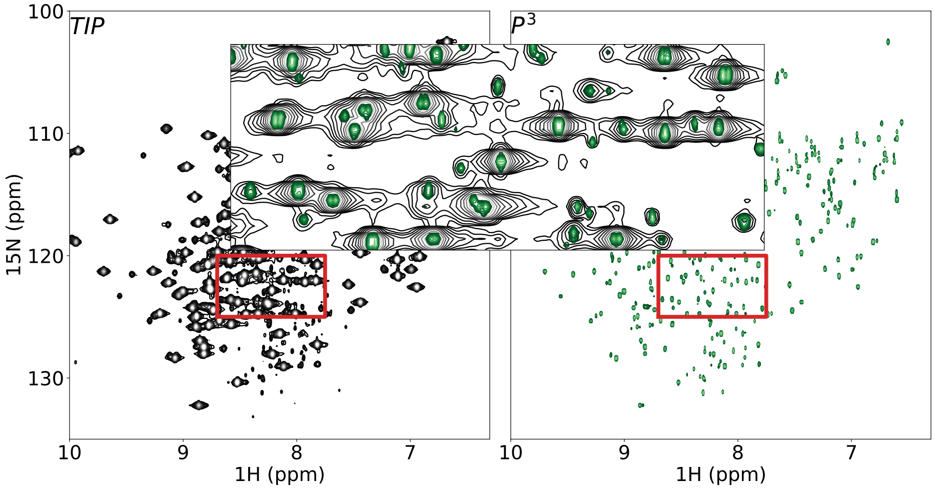

Figure S3: **2D ^{1}H-^{15}N — HSQC spectrum of Azurin protein.** Traditional intensity presentation, TIP, in black and peak probability presentations, P^3 by using MR-Ai, in green color.

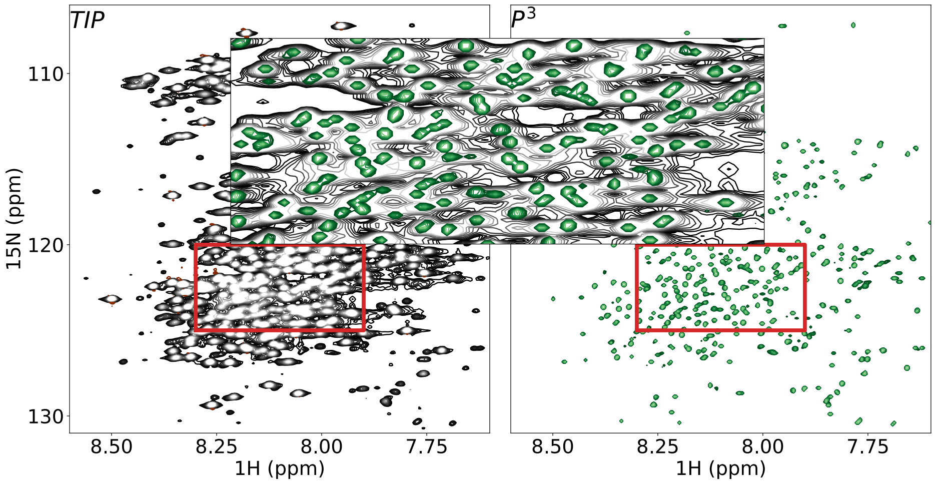

Figure S4: **2D ^{1}H-^{15}N — TROSY spectrum of Tau protein.** Traditional intensity presentation, TIP, in black and peak probability presentations, P^3 by using MR-Ai, in green color.

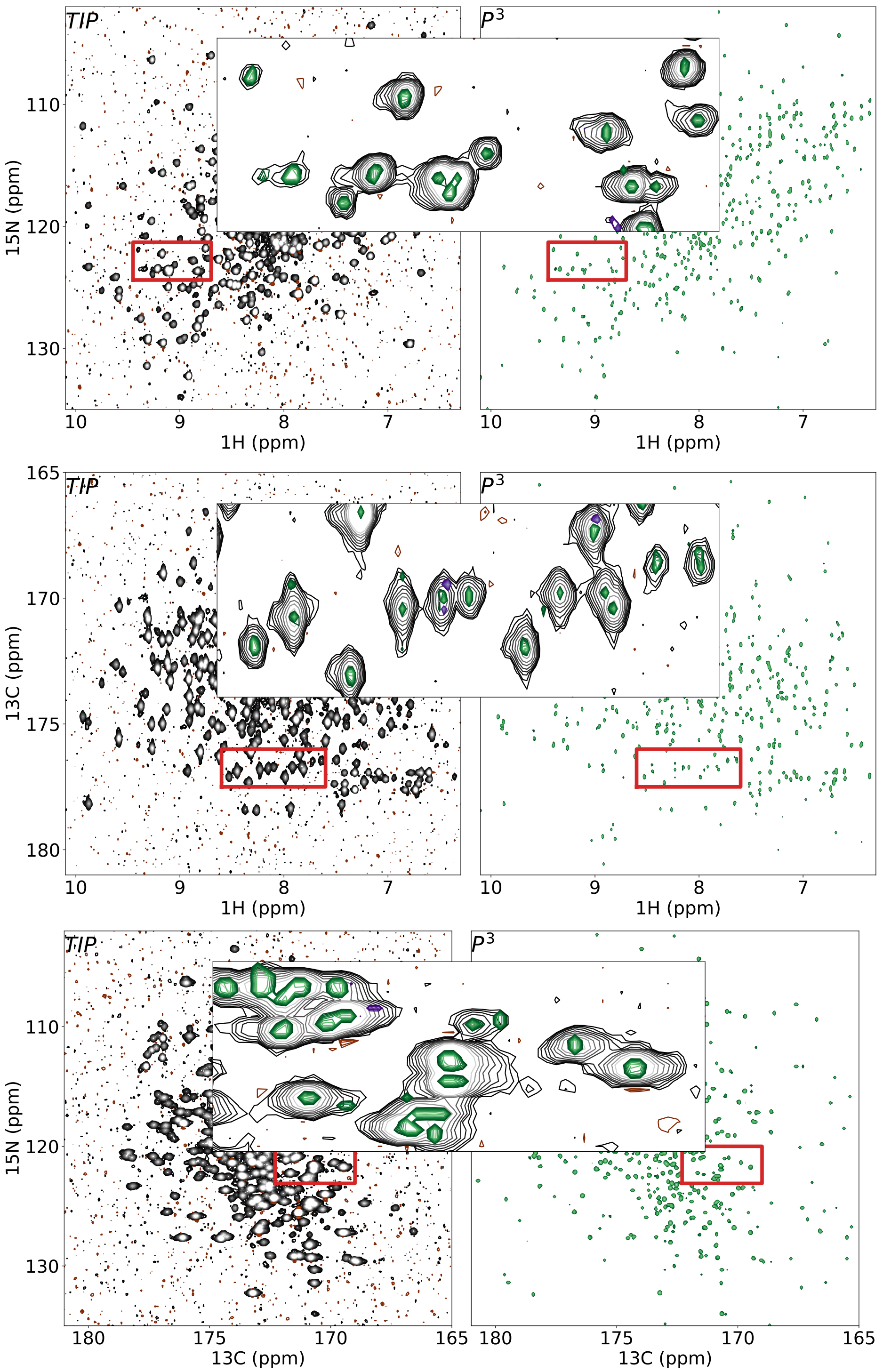

Figure S5: **3D HNCO NUS spectrum of MALT1 protein reconstructed with CS-IST.** Three orthogonal 2D spectrum projections are shown in the intensity presentation (TIP) in black (orange for negative) and in the corresponding peak probability presentation (P^3), generated using MR-Ai, in green (purple for negative).

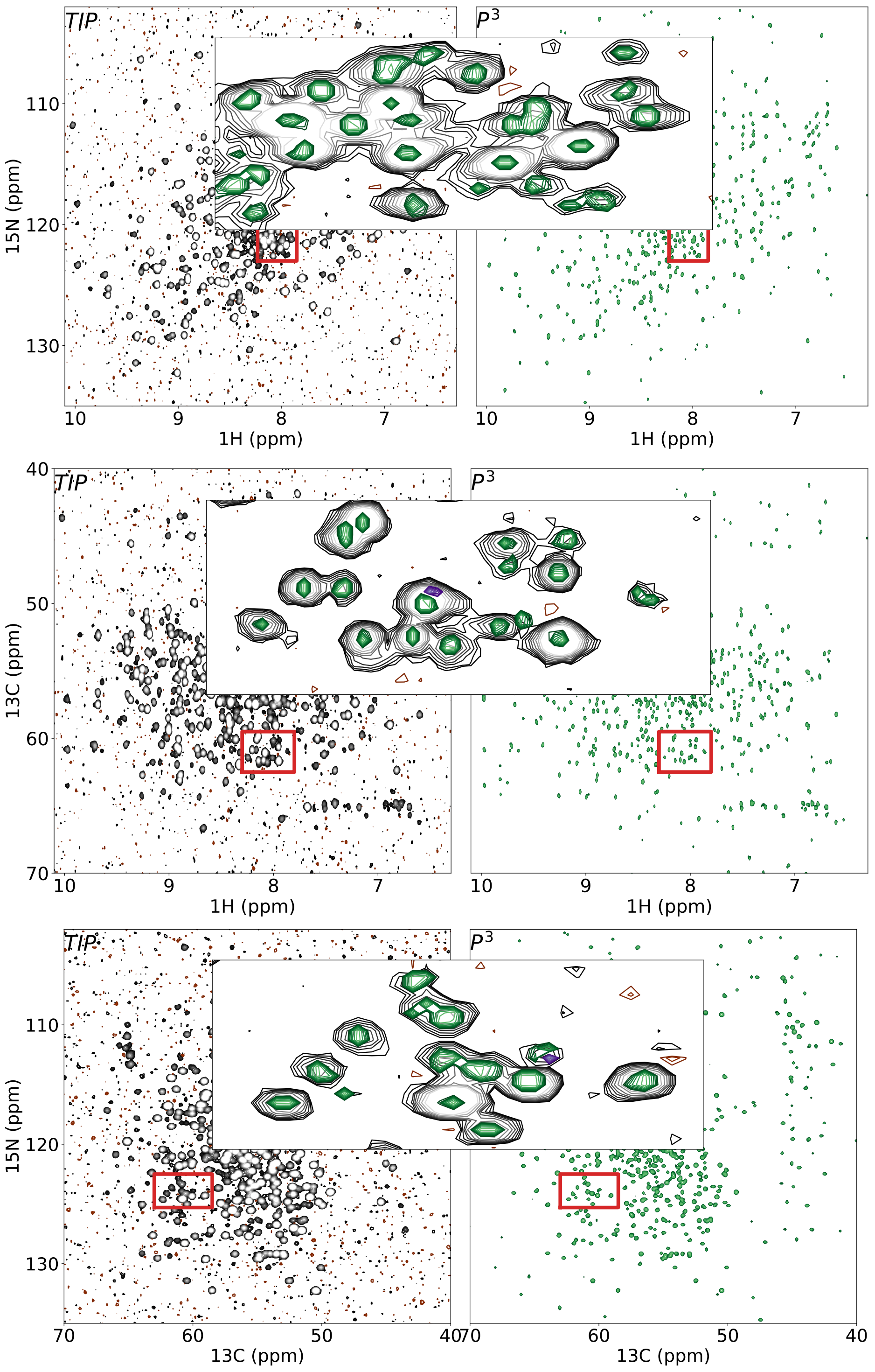

Figure S6: **3D HNCA NUS spectrum of MALT1 reconstructed with CS-IST.** Three orthogonal 2D spectrum projections are shown in the intensity presentation (TIP) in black (orange for negative) and in the corresponding peak probability presentation (P^3), generated using MR-Ai, in green (purple for negative).

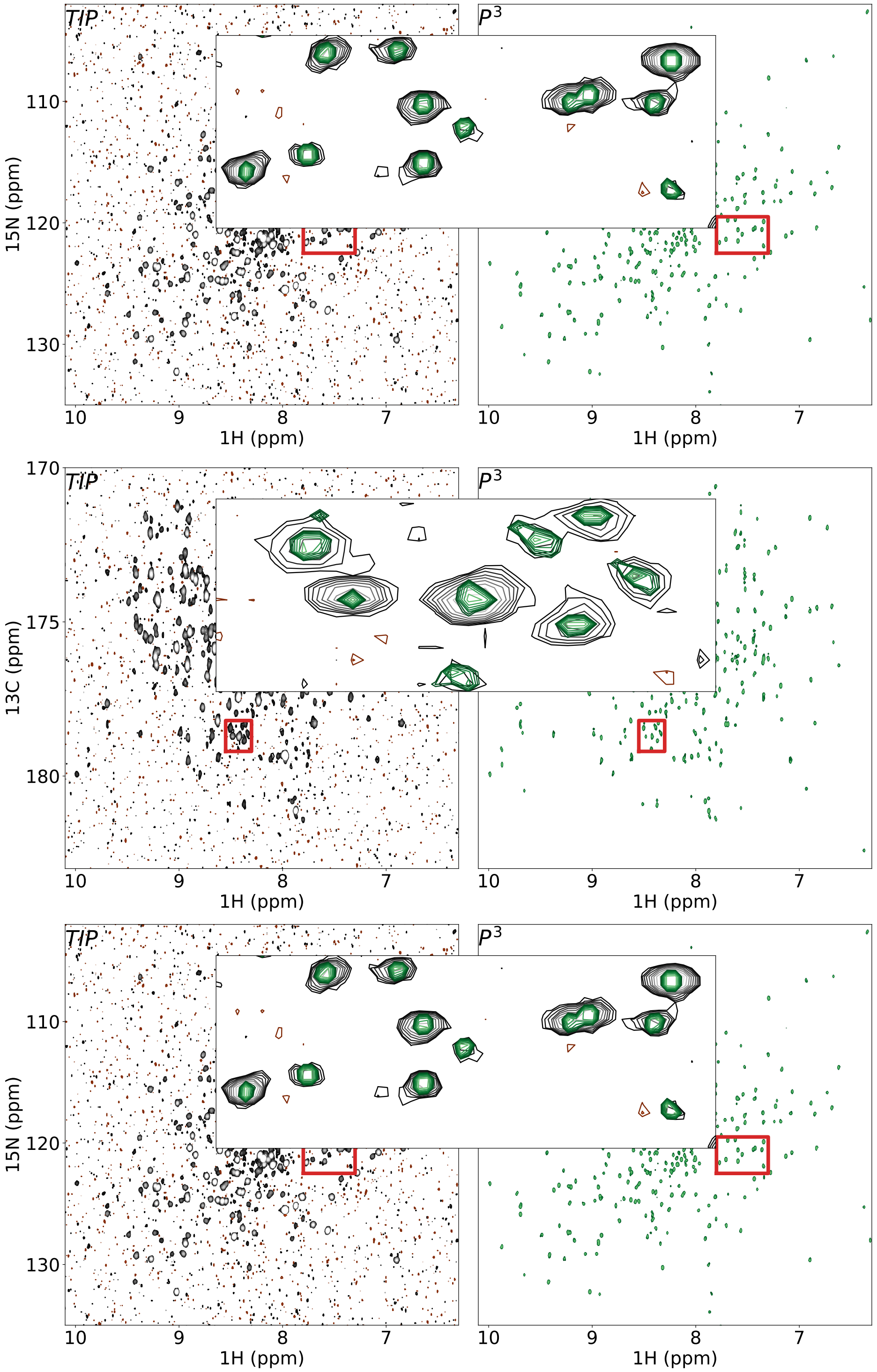

Figure S7: **3D HN(CA)CO NUS spectrum of MALT1 protein reconstructed with CS-IST.** Three orthogonal 2D spectrum projections are shown in the intensity presentation (TIP) in black (orange for negative) and in the corresponding peak probability presentation (P^3), generated using MR-Ai, in green (purple for negative).

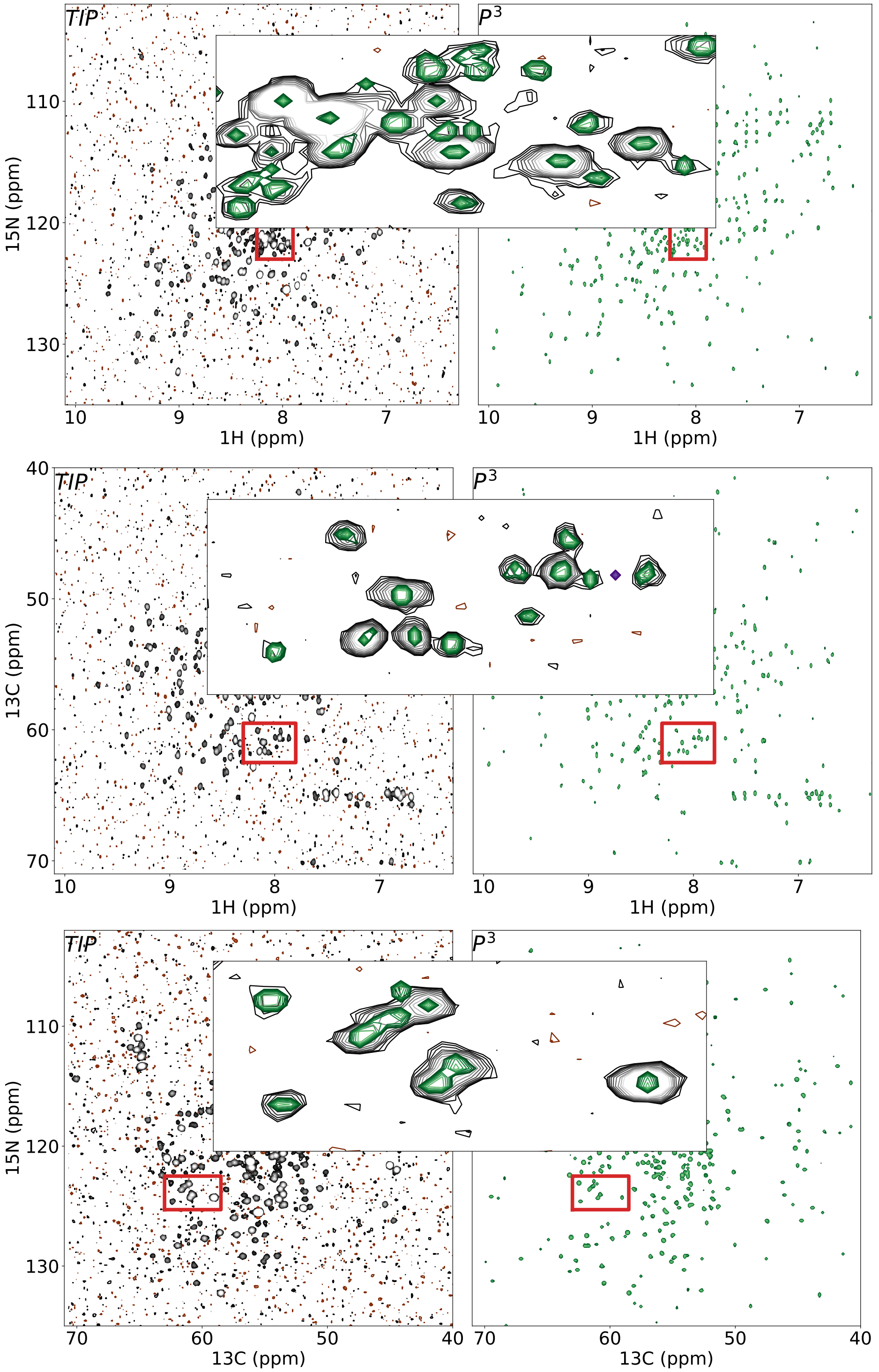

Figure S8: **3D HN(CO)CA NUS spectrum of MALT1 protein reconstructed with CS-IST.** Three orthogonal 2D spectrum projections are shown in the intensity presentation (TIP) in black (orange for negative) and in the corresponding peak probability presentation (P^3), generated using MR-Ai, in green (purple for negative).

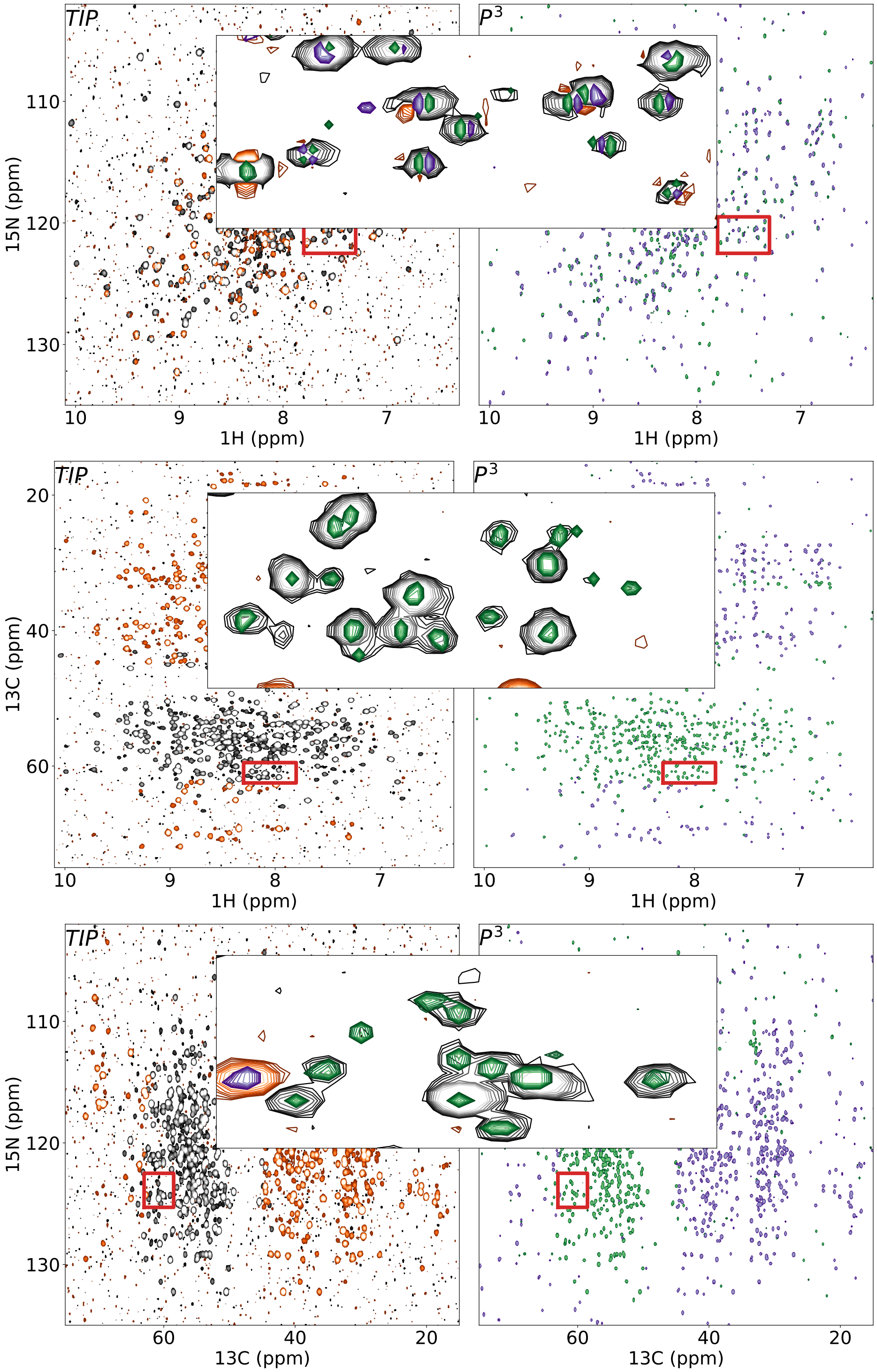

Figure S9: **3D HNCACB NUS spectrum of MALT1 protein reconstructed with CS-IST.** Three orthogonal 2D spectrum projections are shown in the intensity presentation (TIP) in black (orange for negative) and in the corresponding peak probability presentation (P^3), generated using MR-Ai, in green (purple for negative).

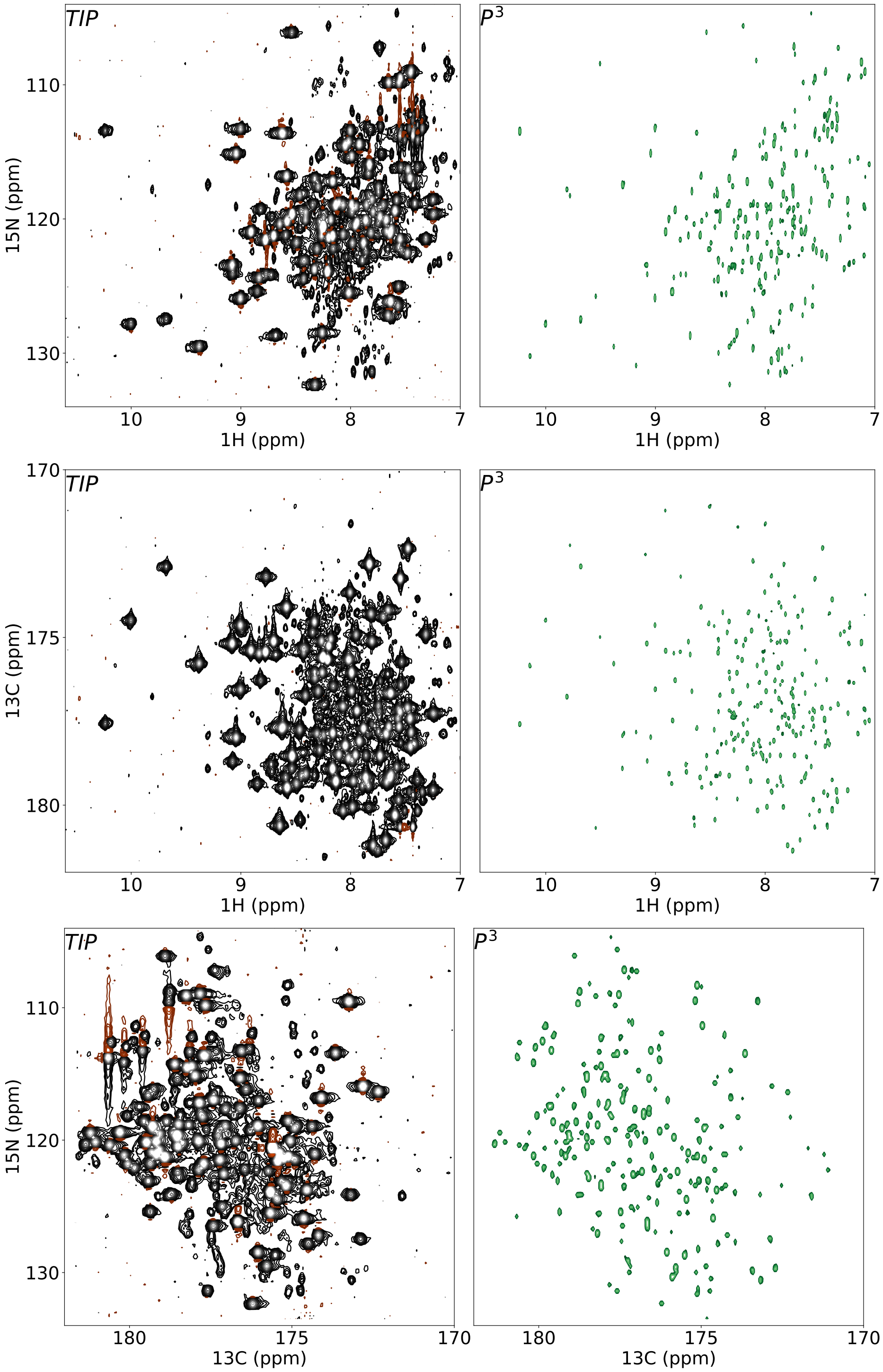

Figure S10: **3D HNCO NUS spectrum of Calmodulin protein reconstructed with CS-IST.** Three orthogonal 2D spectrum projections are shown in the intensity presentation (TIP) in black (orange for negative) and in the corresponding peak probability presentation (P^3), generated using MR-Ai, in green (purple for negative). Note a small phase error in ^{15}N dimension, which is however within the training parameter limits (Table 1) and does not affect the resulting P^3.

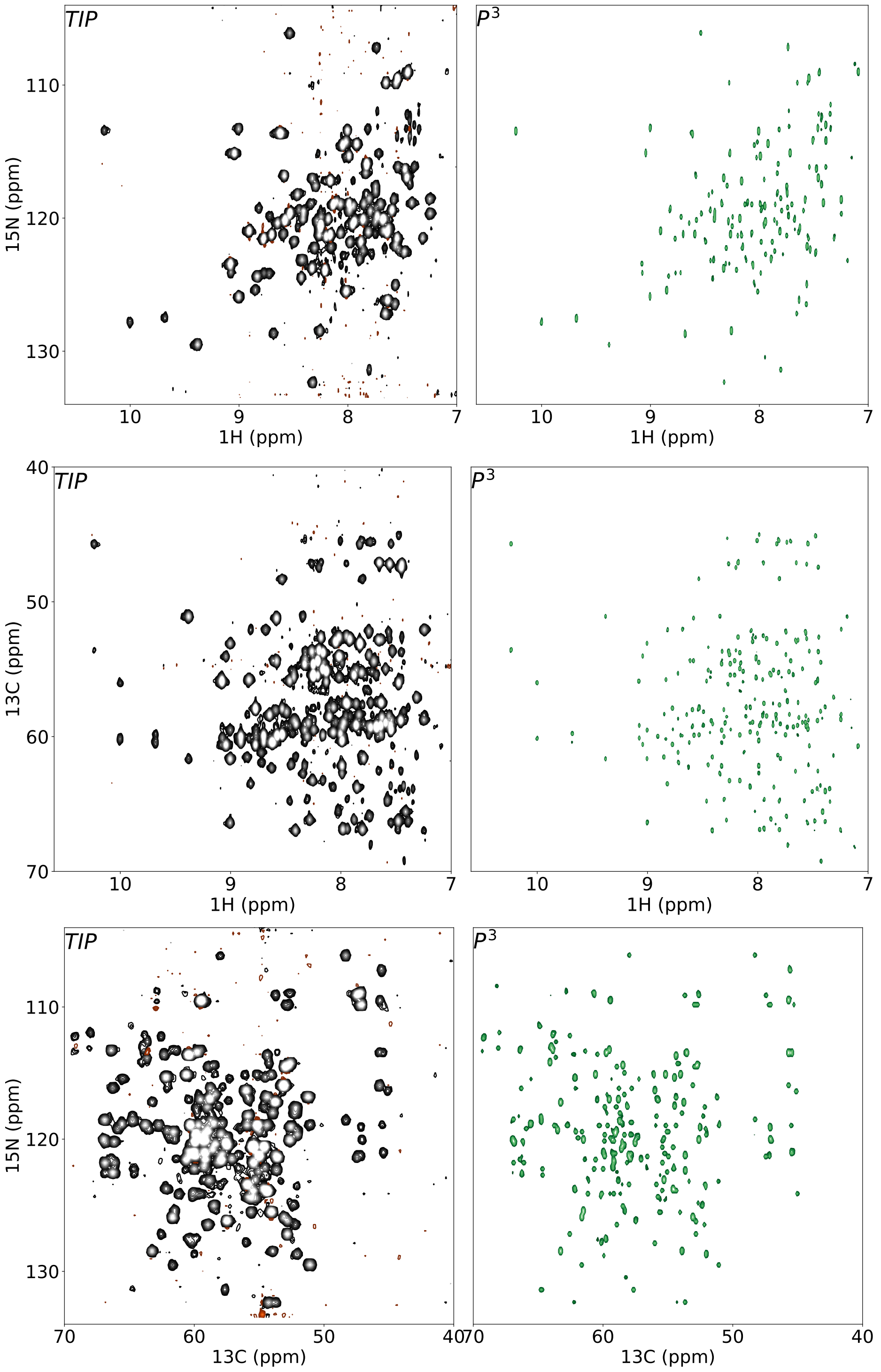

Figure S11: **3D HNCA NUS spectrum of Calmodulin protein reconstructed with CS-IST.** Three orthogonal 2D spectrum projections are shown in the intensity presentation (TIP) in black (orange for negative) and in the corresponding peak probability presentation (P^3), generated using MR-Ai, in green (purple for negative).

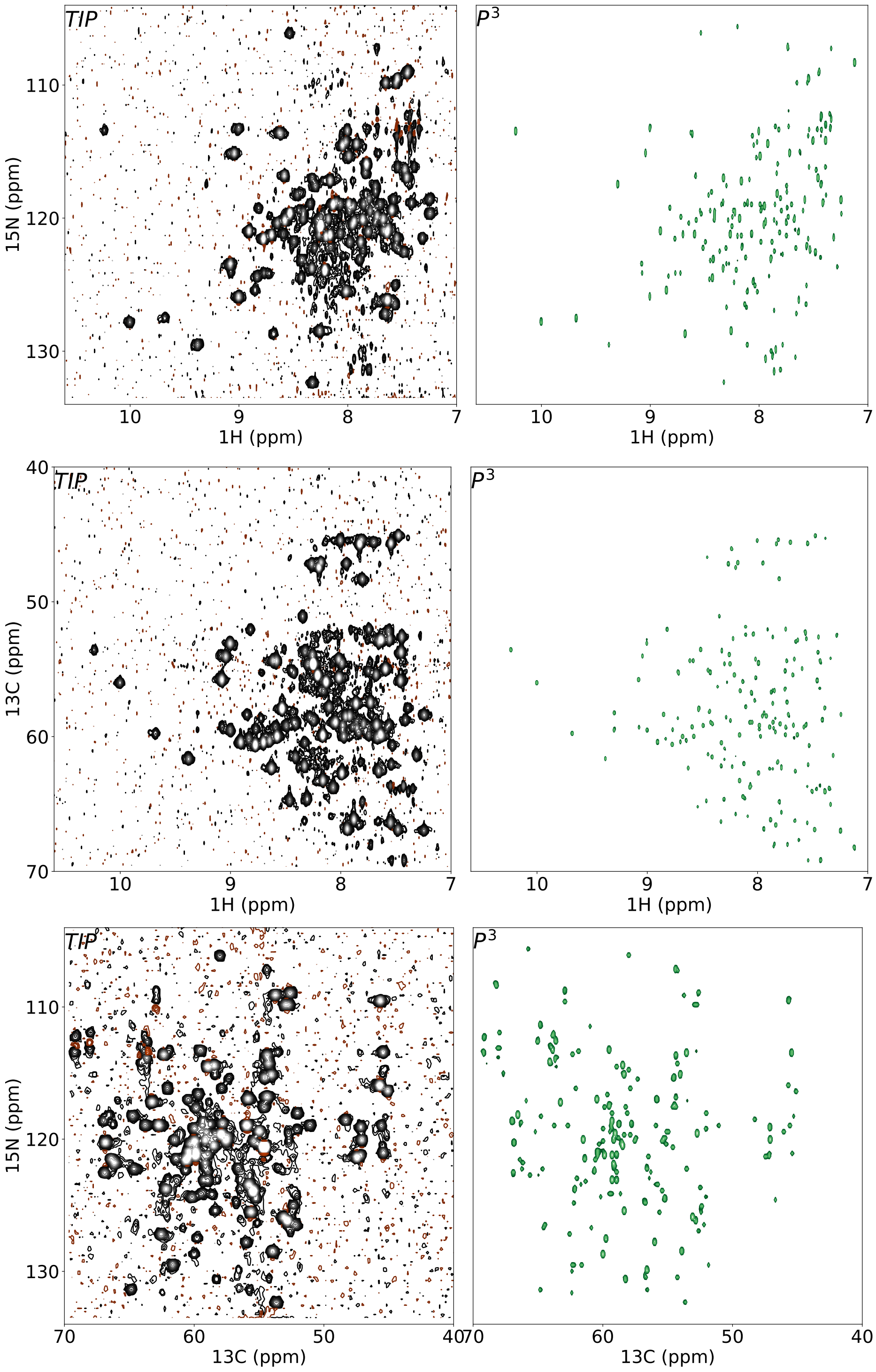

Figure S12: **3D HN(CO)CA NUS spectrum of Calmodulin protein reconstructed with CS-IST.** Three orthogonal 2D spectrum projections are shown in the intensity presentation (TIP) in black (orange for negative) and in the corresponding peak probability presentation (P^3), generated using MR-Ai, in green (purple for negative).

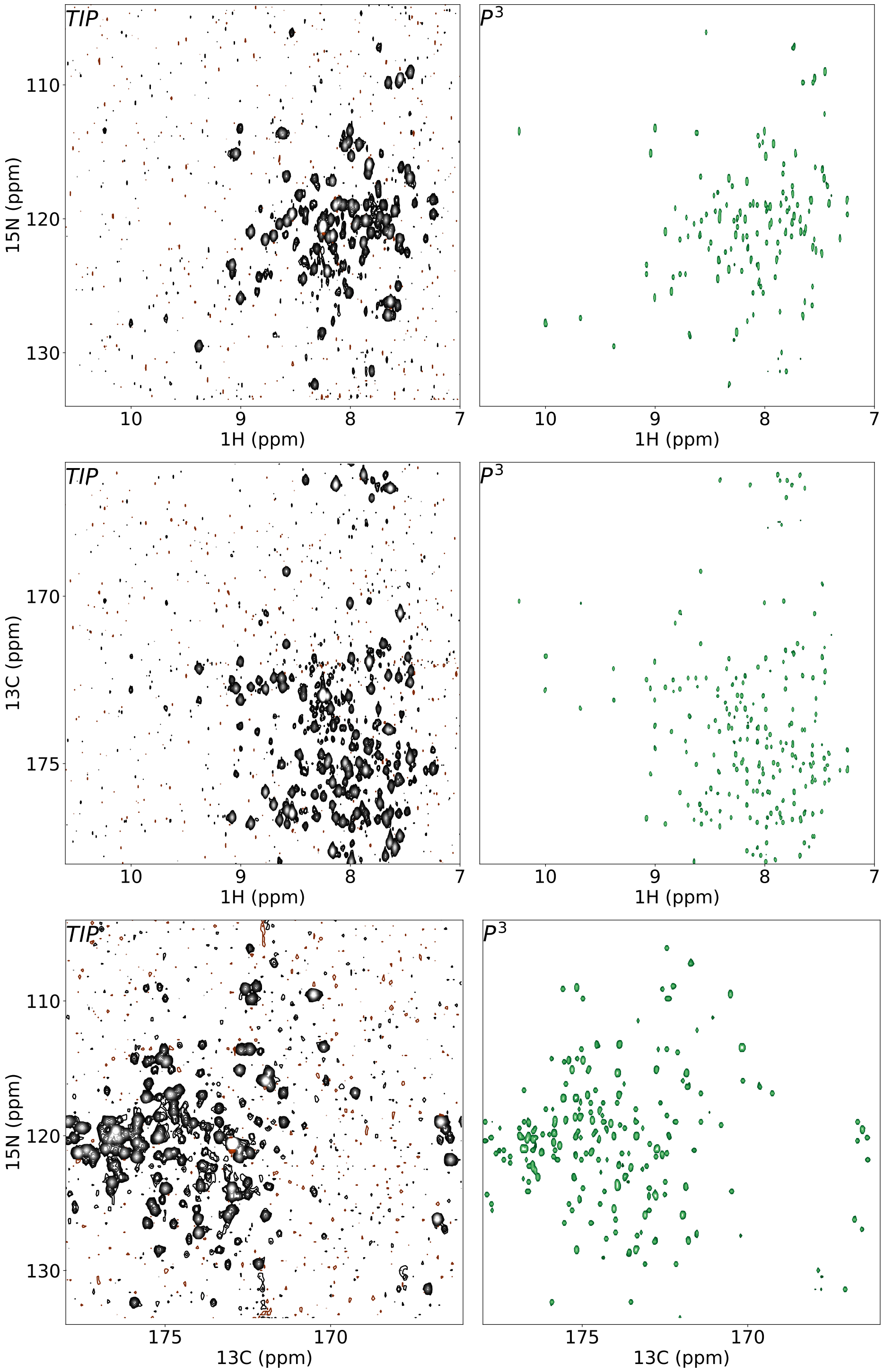

Figure S13: **3D HN(CA)CO NUS spectrum of Calmodulin protein reconstructed with CS-IST.** Three orthogonal 2D spectrum projections are shown in the intensity presentation (TIP) in black (orange for negative) and in the corresponding peak probability presentation (P^3), generated using MR-Ai, in green (purple for negative).

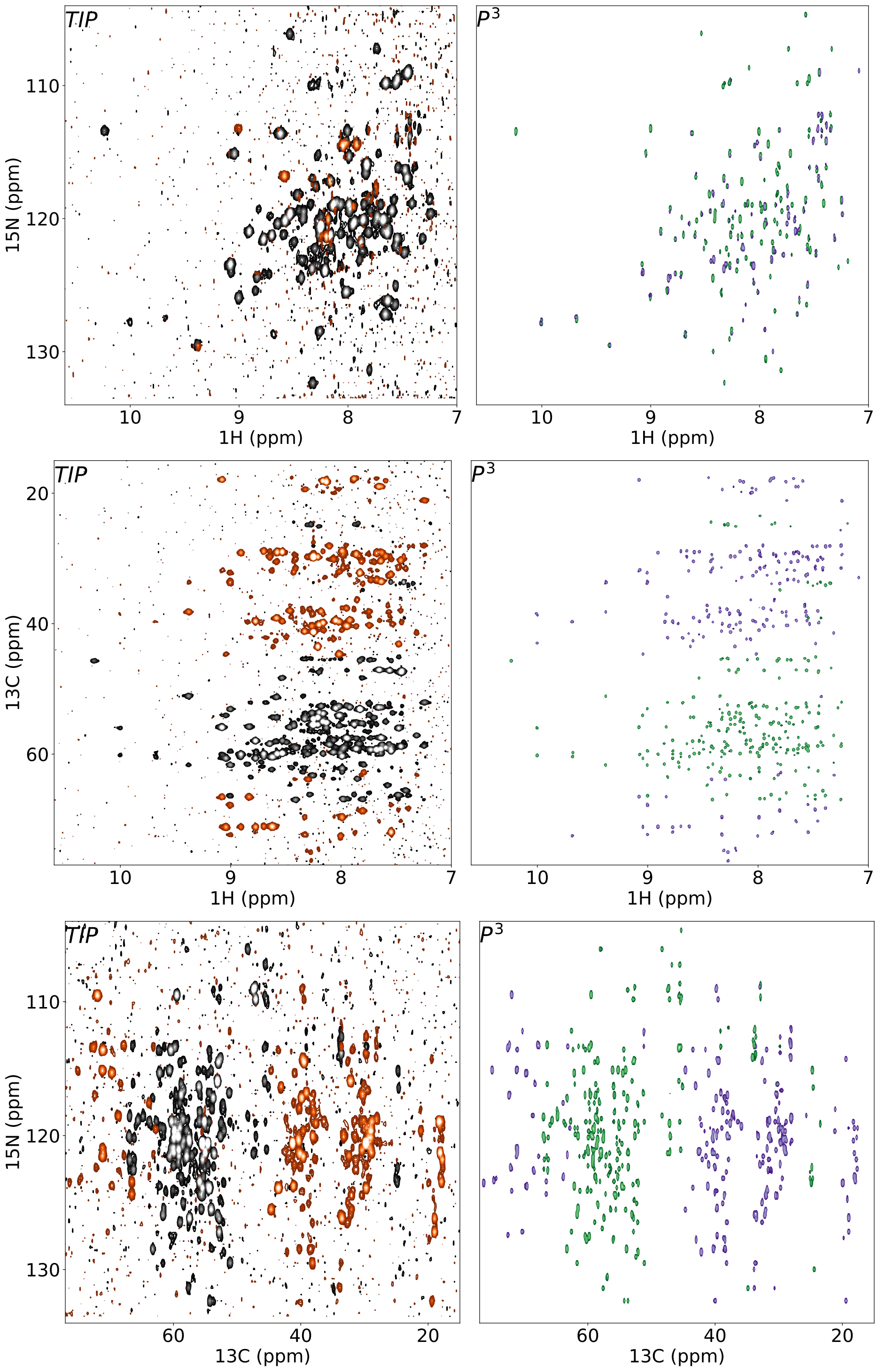

Figure S14: **3D HNCACB NUS spectrum of Calmodulin protein reconstructed with CS-IST.** Three orthogonal 2D spectrum projections are shown in the intensity presentation (TIP) in black (orange for negative) and in the corresponding peak probability presentation (P^3), generated using MR-Ai, in green (purple for negative).

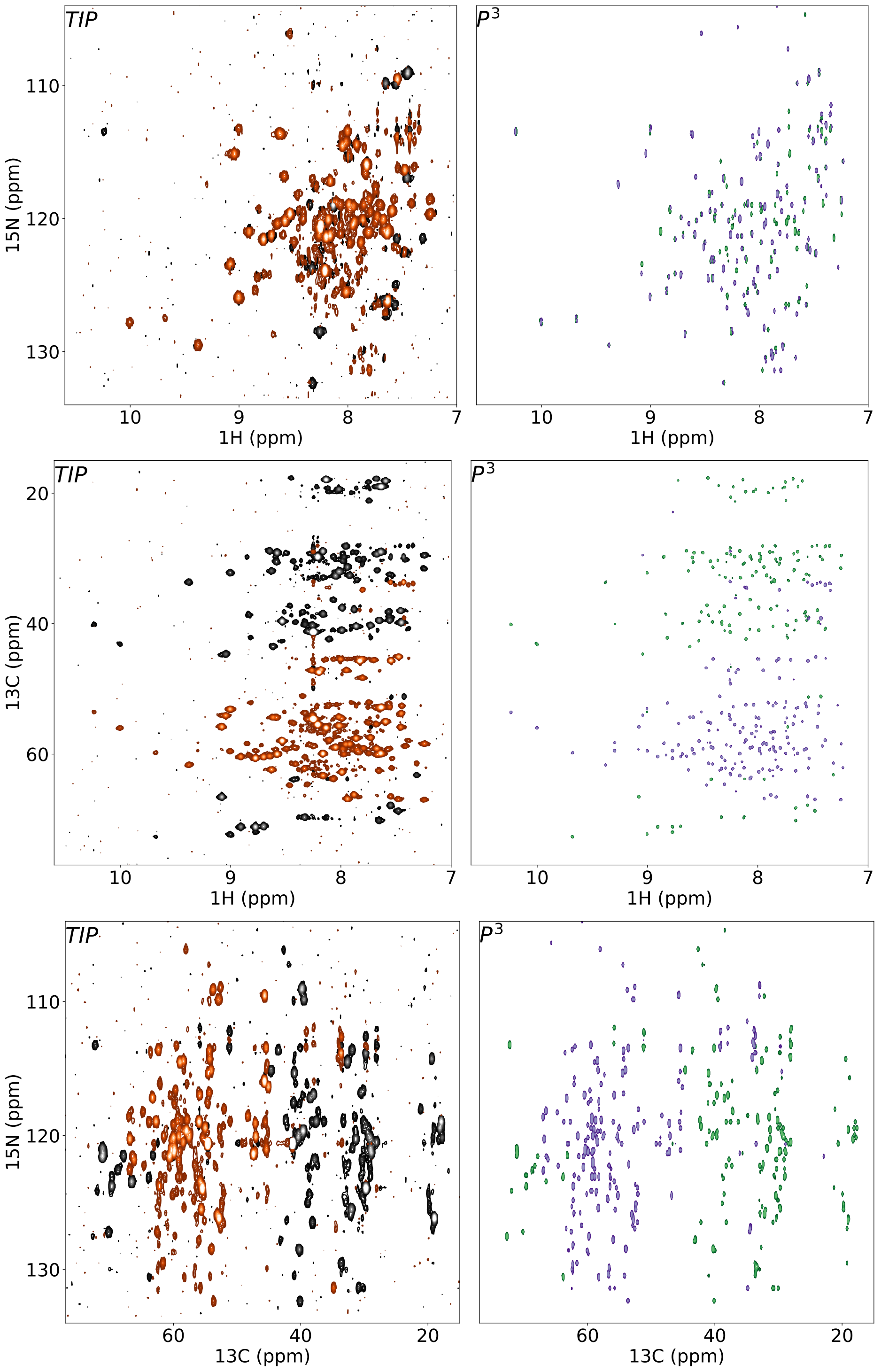

Figure S15: **3D HN(CO)CACB NUS spectrum of Calmodulin protein reconstructed with CS-IST.** Three orthogonal 2D spectrum projections are shown in the intensity presentation (TIP) in black (orange for negative) and in the corresponding peak probability presentation (P^3), generated using MR-Ai, in green (purple for negative).

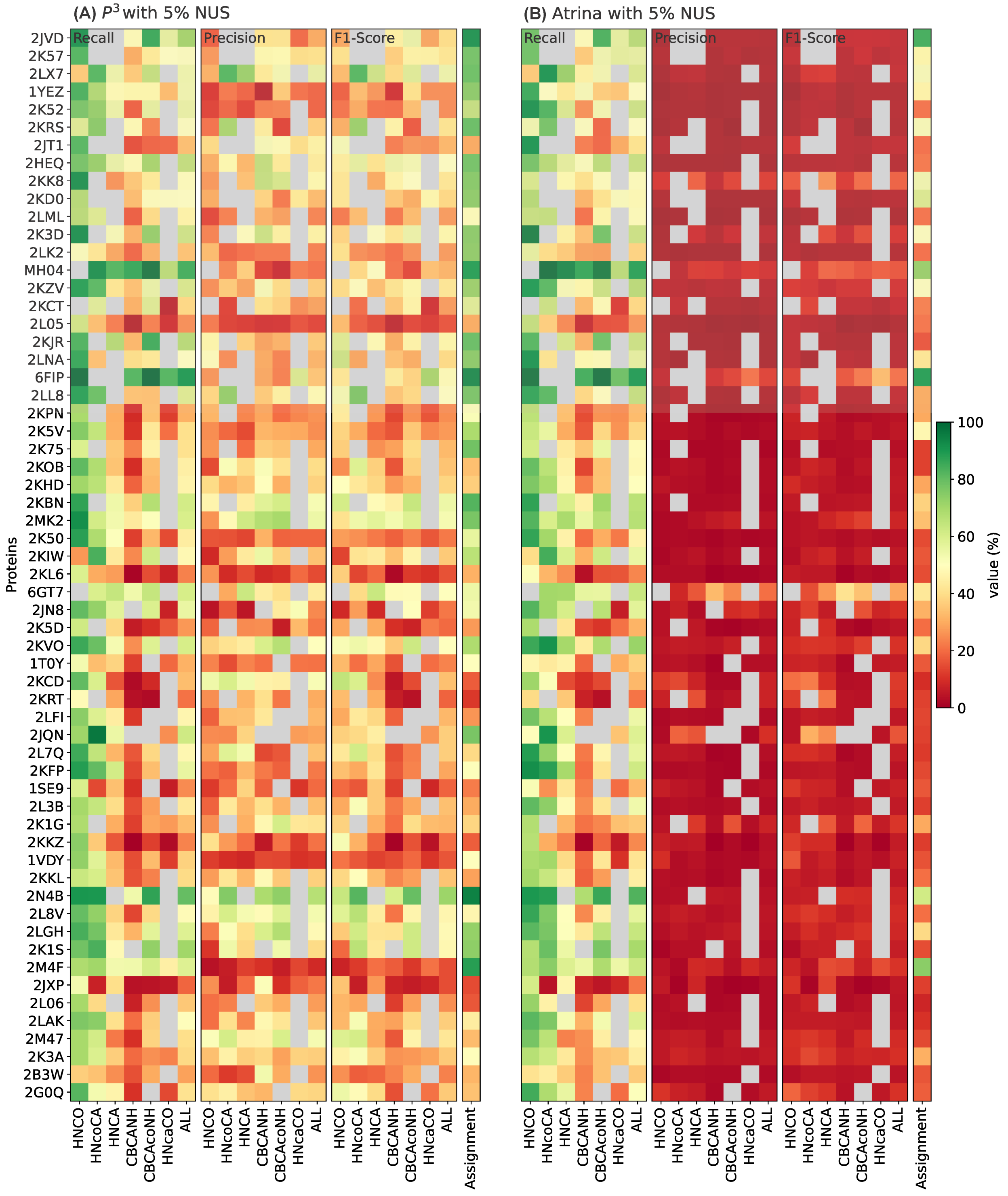

Figure S16: **Performance comparison of MR-Ai and ARTINA at 5% NUS across all 60 proteins.** Heatmaps show recall, precision, F1-score, and CYANA assignment accuracy for six backbone experiments. Grey cells indicate missing spectra. MR-Ai maintains substantially higher precision and F1-scores than ARTINA at this extreme undersampling level.

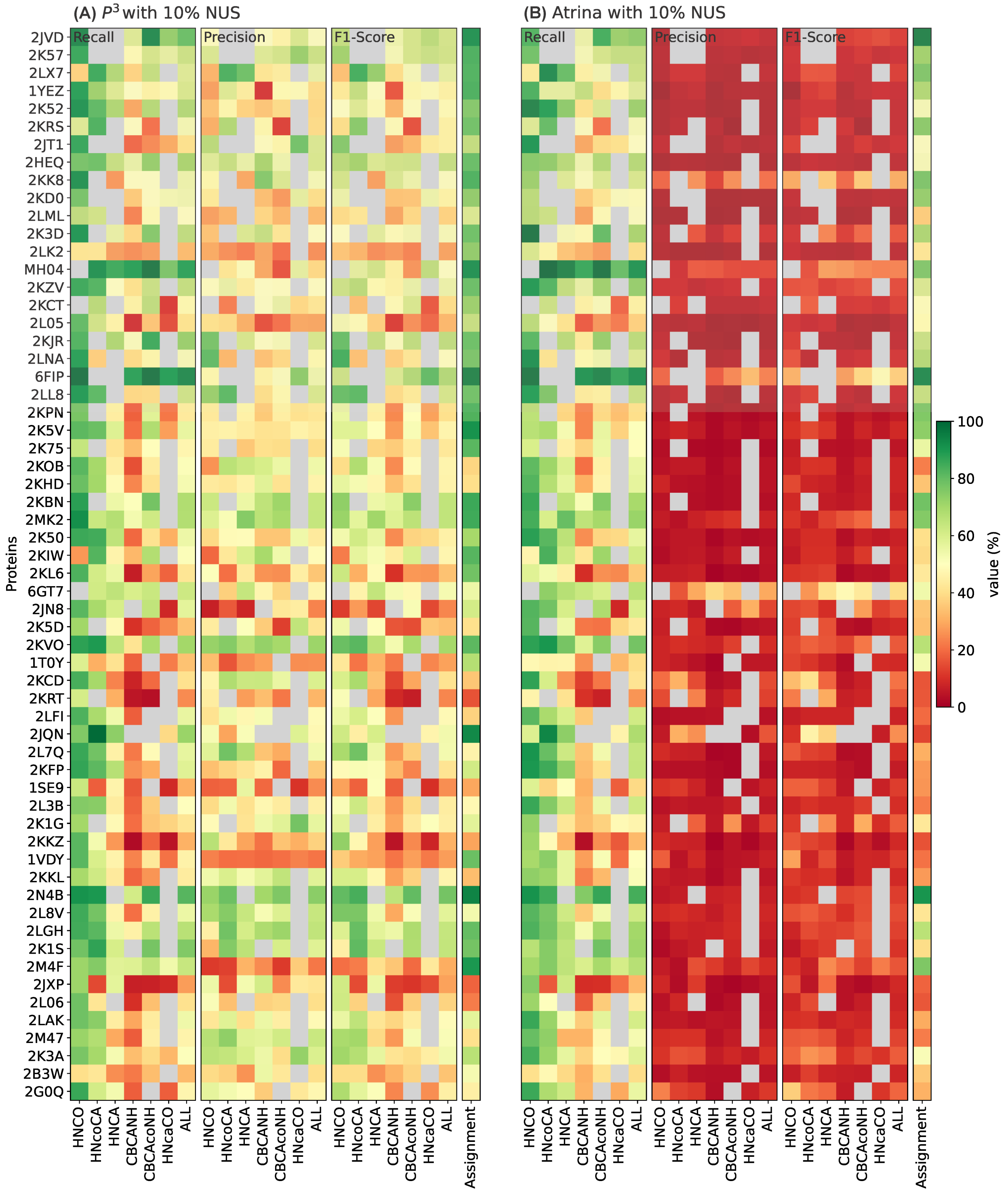

Figure S17: **Performance comparison of MR-Ai and ARTINA at 10% NUS across all 60 proteins.** MR-Ai preserves high precision and F1-scores across experiments and proteins, while ARTINA performance degrades sharply under NUS conditions.

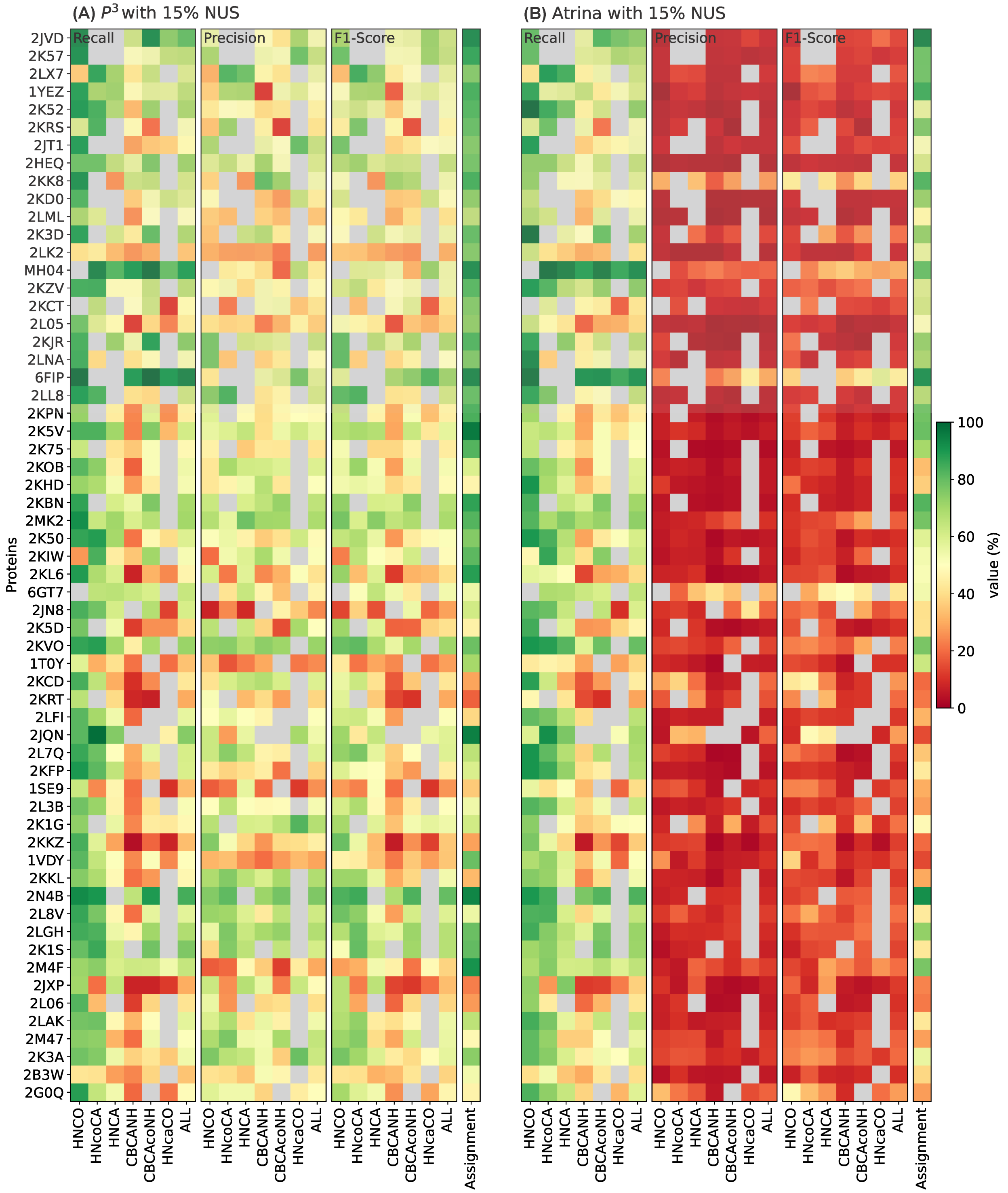

Figure S18: **Performance comparison of MR-Ai and ARTINA at 15% NUS across all 60 proteins.** MR-Ai continues to outperform ARTINA in precision, F1-score, and assignment accuracy, reflecting higher robustness to moderate undersampling.

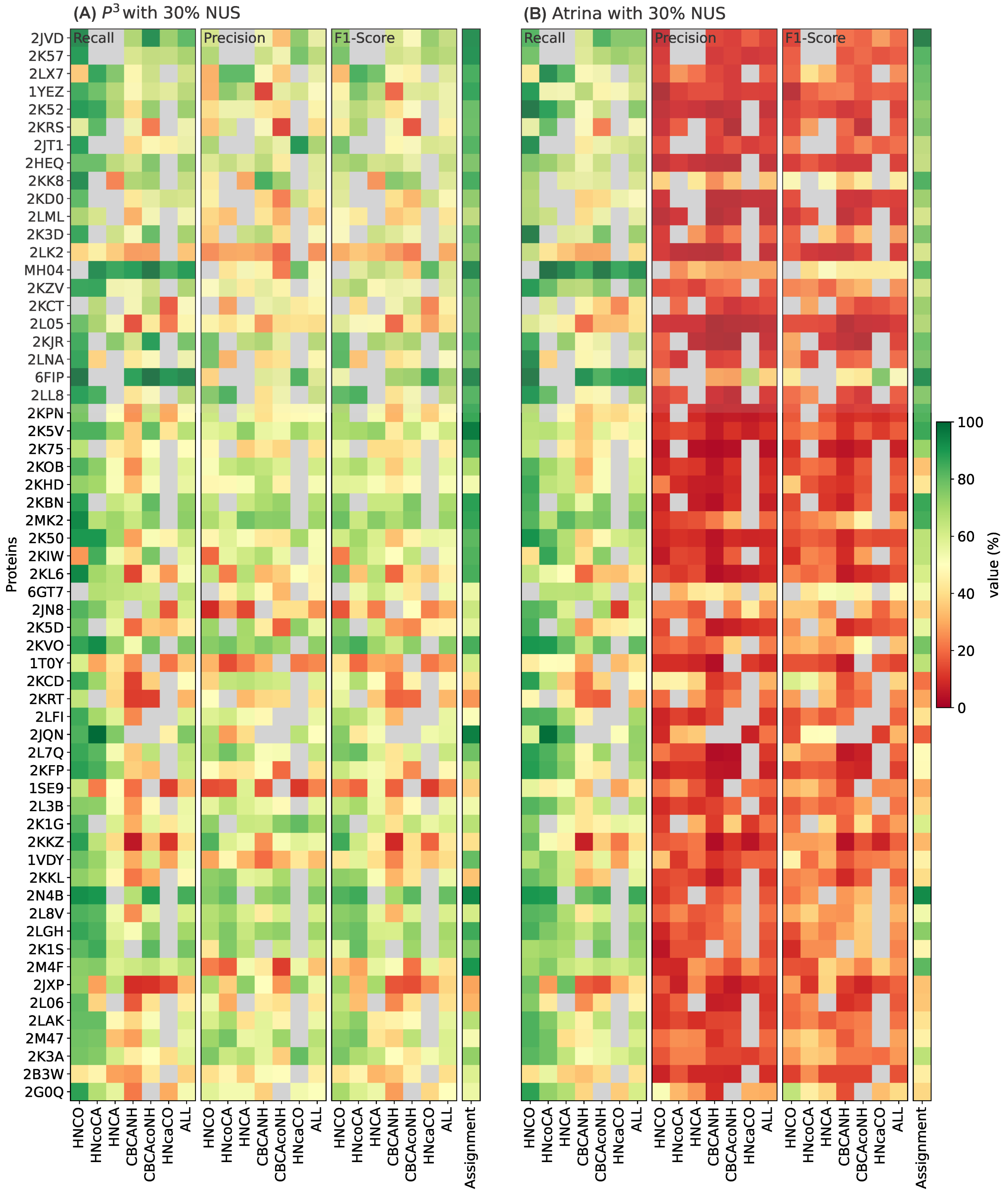

Figure S19: **Performance comparison of MR-Ai and ARTINA at 30% NUS across all 60 proteins.** At mid-level sampling, MR-Ai maintains consistently strong peak-picking and assignment performance, whereas ARTINA remains sensitive to NUS-induced artefacts.

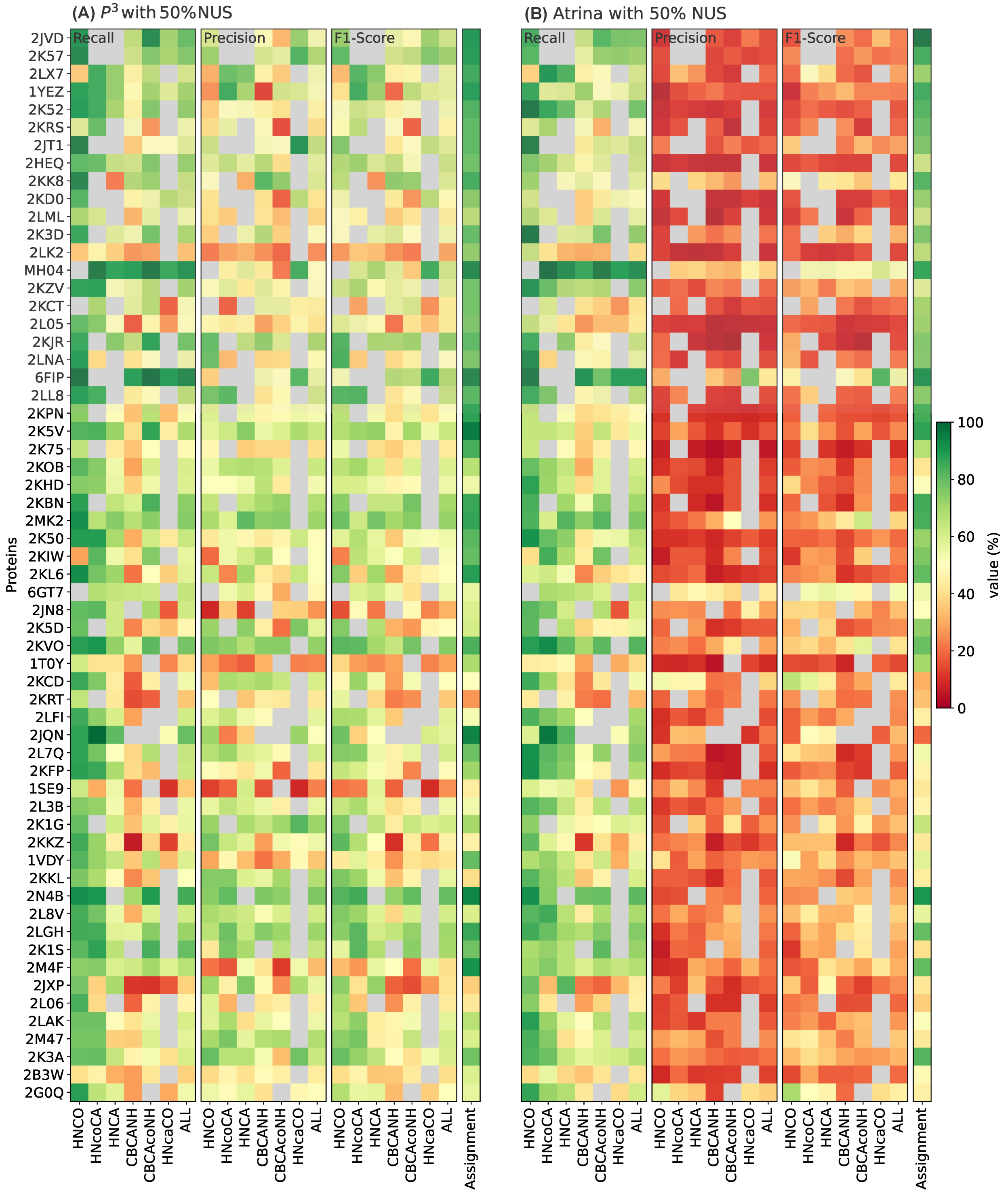

Figure S20: **Performance comparison of MR-Ai and ARTINA at 50% NUS across all 60 proteins.** MR-Ai achieves uniformly high precision and F1-scores, with assignment accuracy comparable to fully sampled data, while ARTINA shows partial recovery but remains less stable.

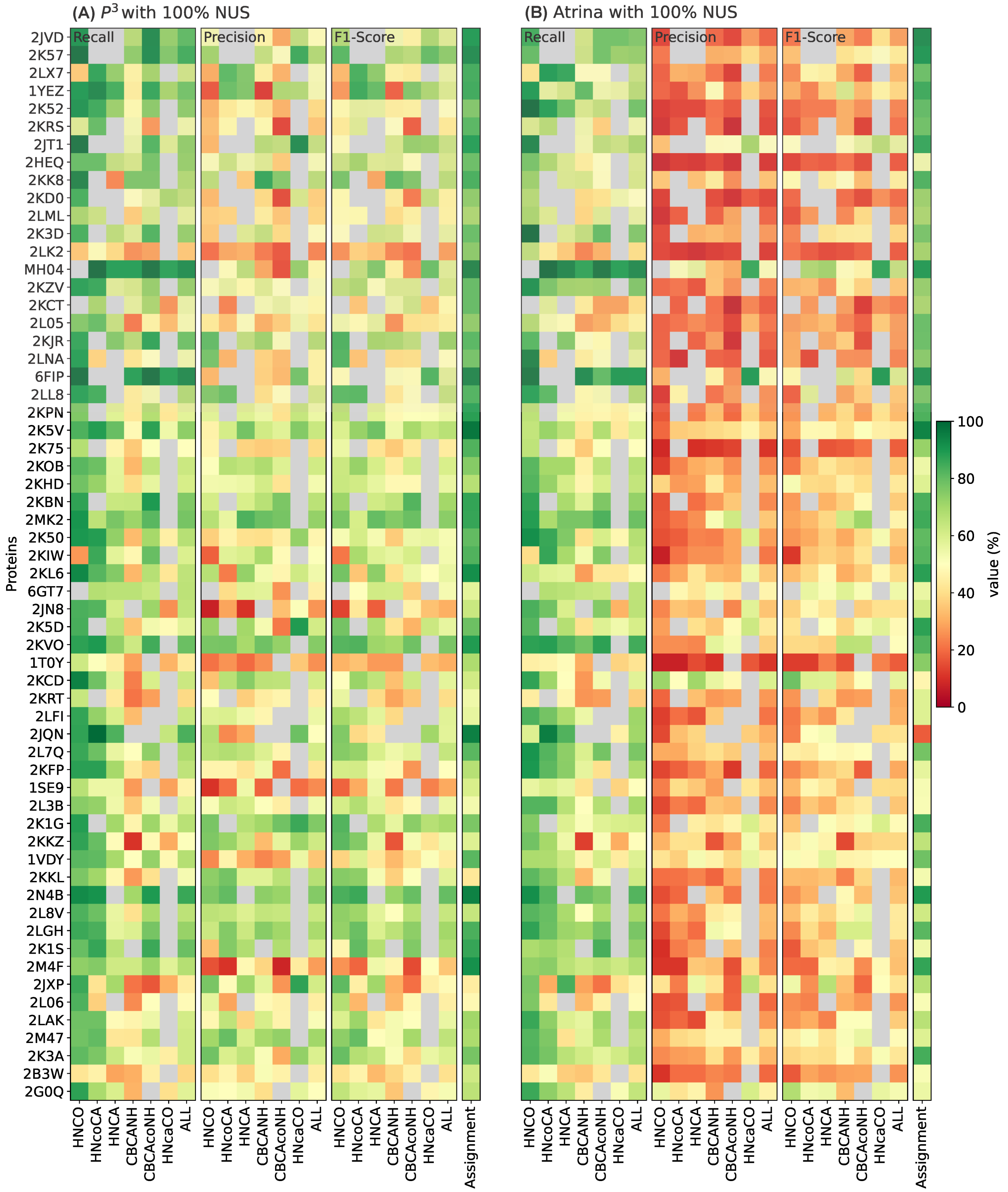

Figure S21: **Performance comparison of MR-Ai and ARTINA for fully sampled spectra across all 60 proteins.** Both methods achieve high recall, but MR-Ai provides consistently higher precision and F1-scores across all backbone experiments.

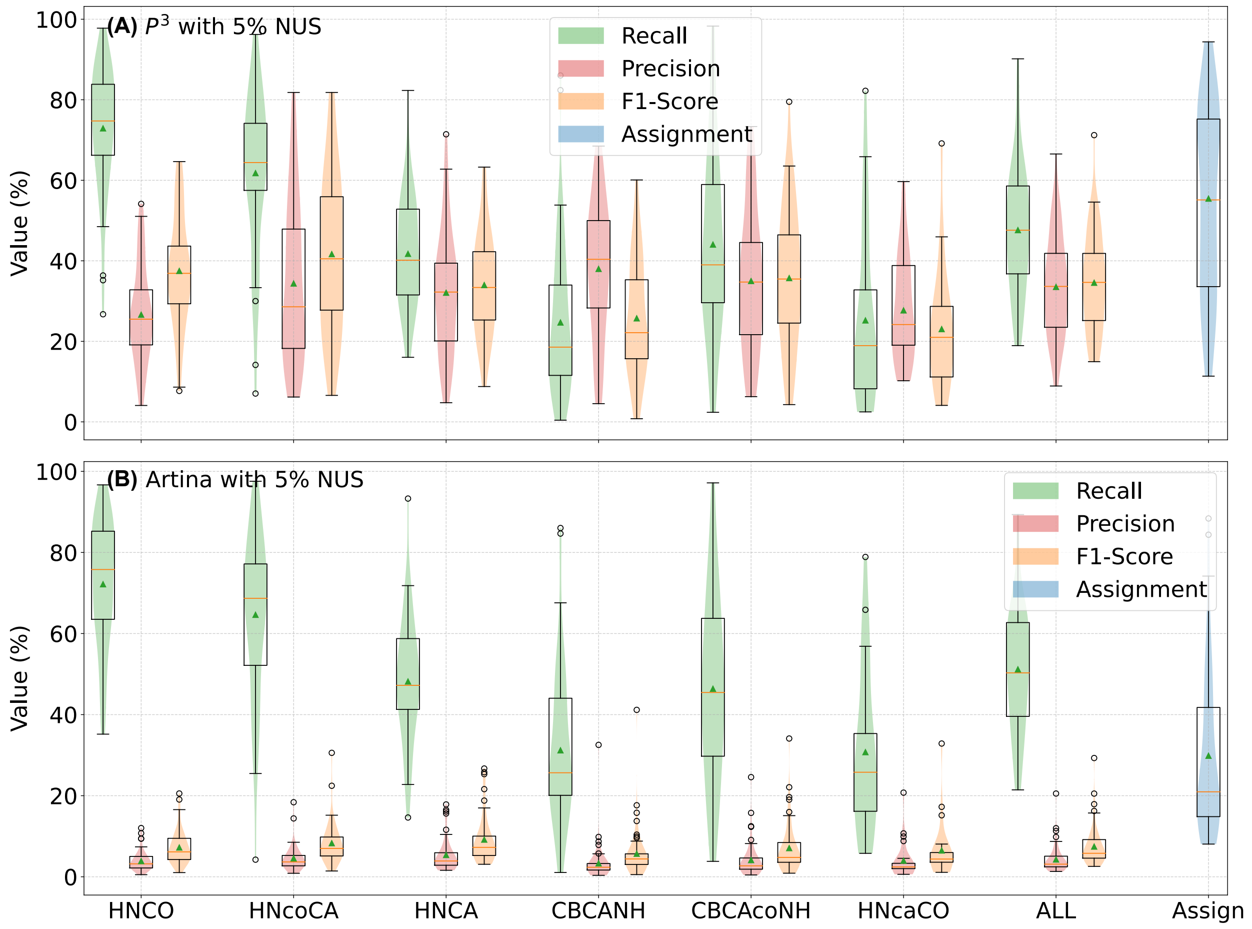

Figure S22: **Peak-picking and assignment performance at 5% NUS across all 60 proteins.** Violin/box plots show the distribution of recall, precision, F1-score, and CAYANA assignment accuracy for P^3 (top) and ARTINA (bottom) across six backbone experiment types (HNCO, HNcoCA, HNCA, CBCANH, CBCAcoNH, HNcaCO). At this extreme undersampling level, P^3 maintains substantially higher precision and F1-scores, while ARTINA produces large numbers of false positives and near-zero assignment accuracy for many spectra. These results demonstrate the strong robustness of MR-Ai to severe NUS undersampling.

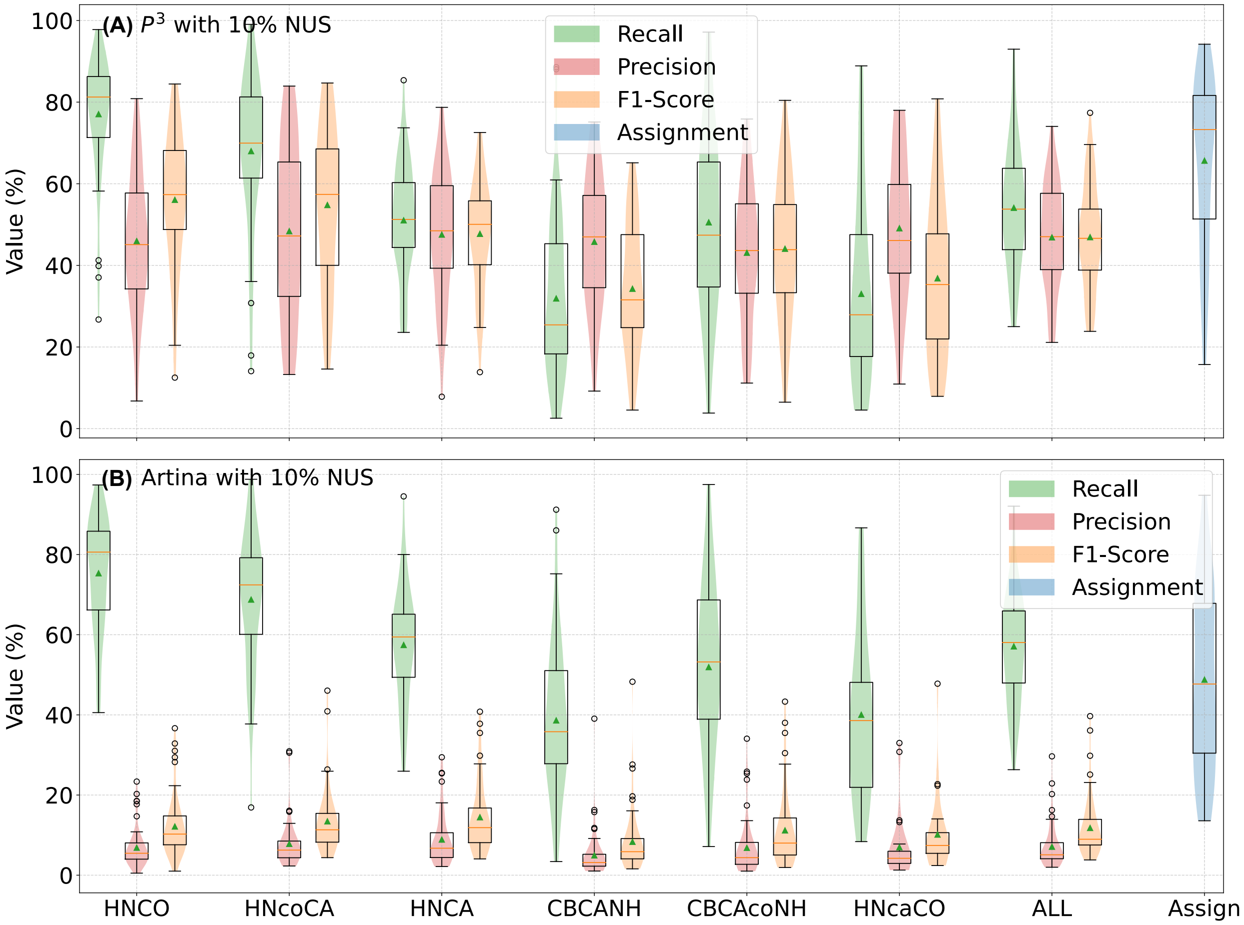

Figure S23: **Peak-picking and assignment performance at 10% NUS across all 60 proteins.** P^3 achieves consistently high precision and F1-scores across experiment types, with CYANA assignments remaining stable and accurate. In contrast, ARTINA exhibits pronounced degradation in precision and assignment accuracy, reflecting its sensitivity to NUS-related reconstruction artefacts. These results show that MR-Ai remains reliable even under strong undersampling conditions relevant for fast 3D NMR acquisition.

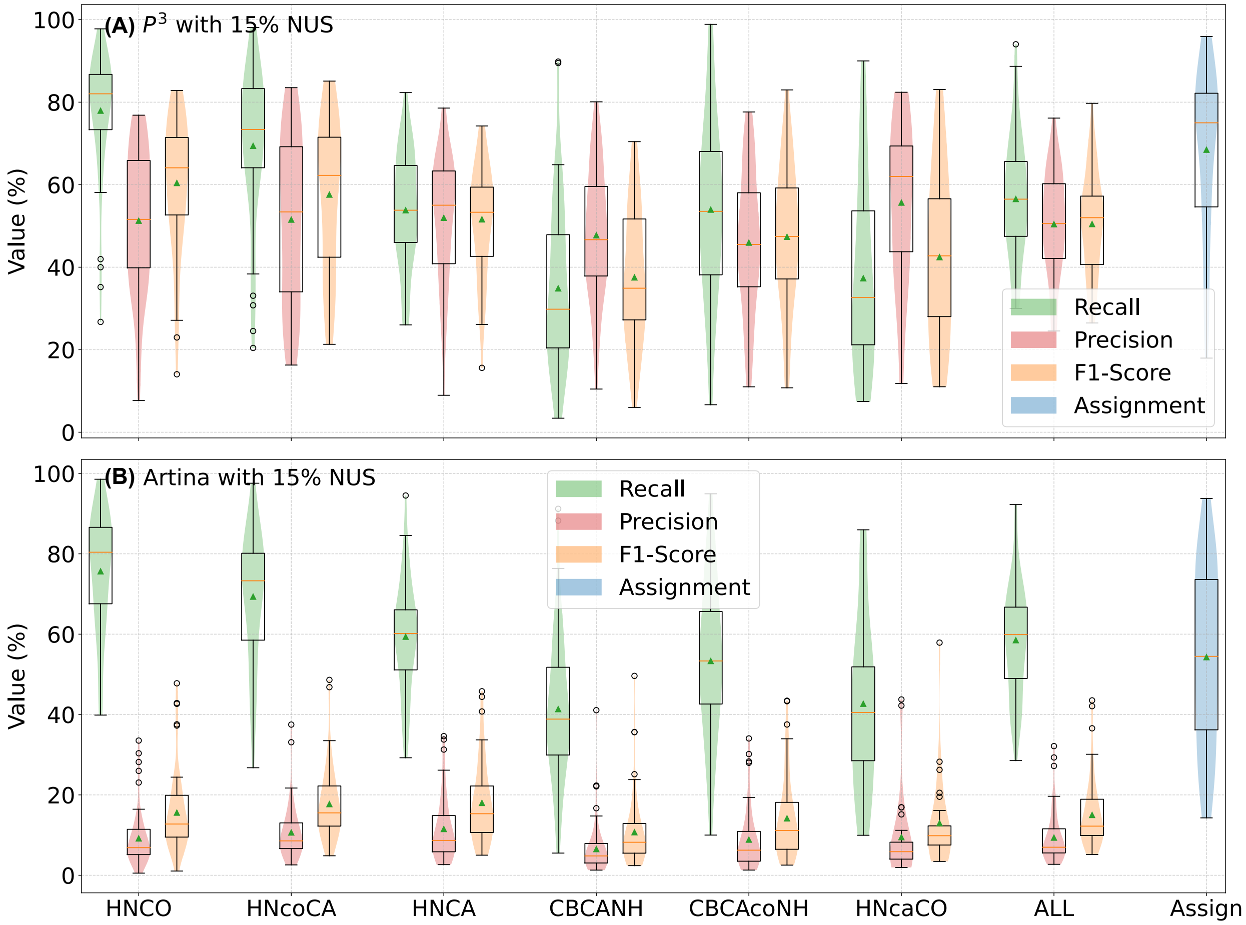

Figure S24: **Peak-picking and assignment performance at 15% NUS across all 60 proteins.** MR-Ai continues to outperform ARTINA across all evaluation metrics, with P^3 yielding higher precision, improved F1-scores, and more accurate CYANA assignments. Although ARTINA shows partial recovery compared to more aggressive NUS levels, its precision and assignment accuracy remain markedly lower than those achieved with P^3. This demonstrates that MR-Ai provides robust peak detection and assignment input at moderate undersampling levels.

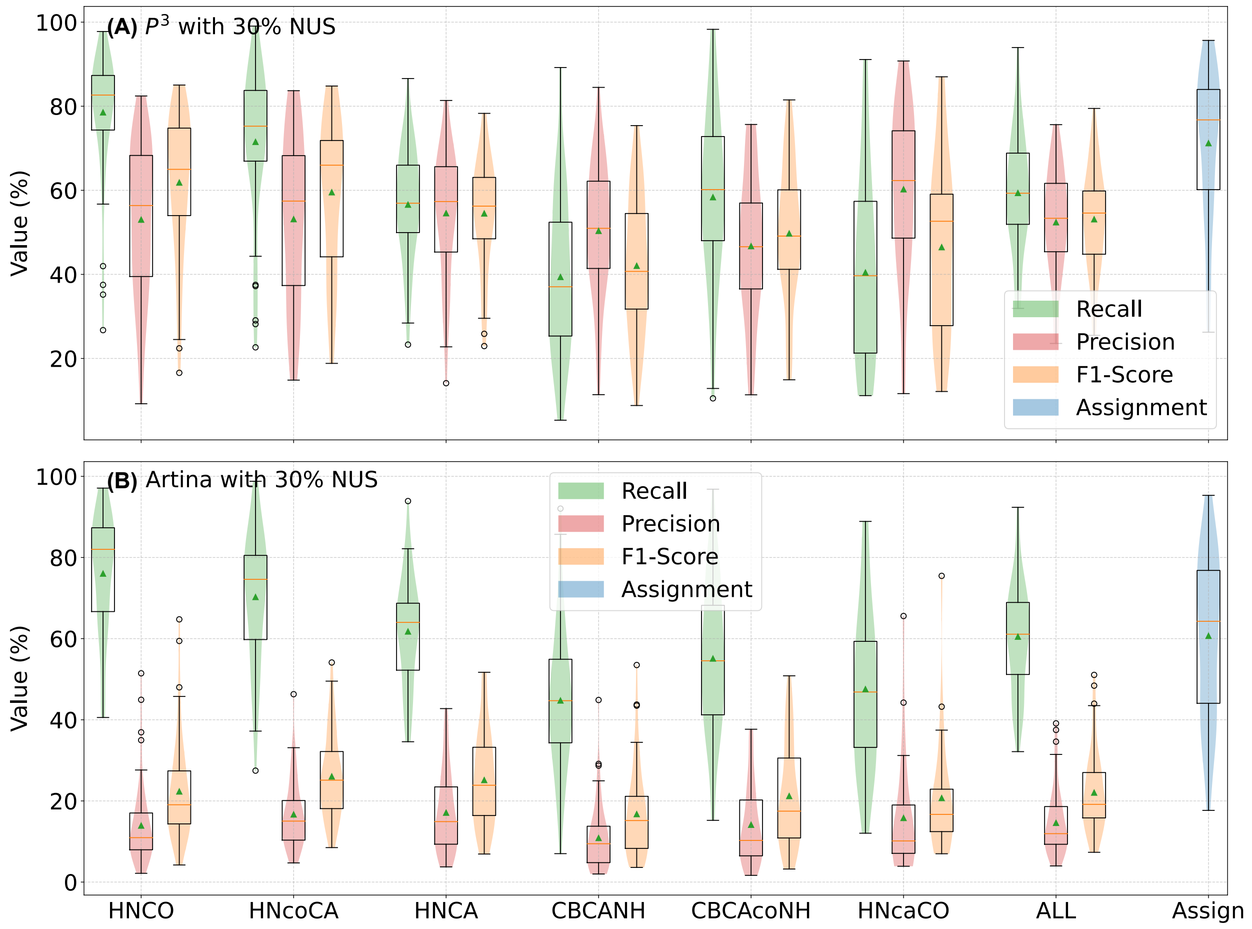

Figure S25: **Peak-picking and assignment performance at 30% NUS across all 60 proteins.** P^3 achieves uniformly strong precision and F1-scores across all experiment types, and CYANA assignments remain close to fully sampled performance. ARTINA remains sensitive to NUS-induced artefacts, showing lower precision and reduced assignment accuracy despite higher sampling density. These results highlight the stability of MR-Ai across mid-range NUS conditions.

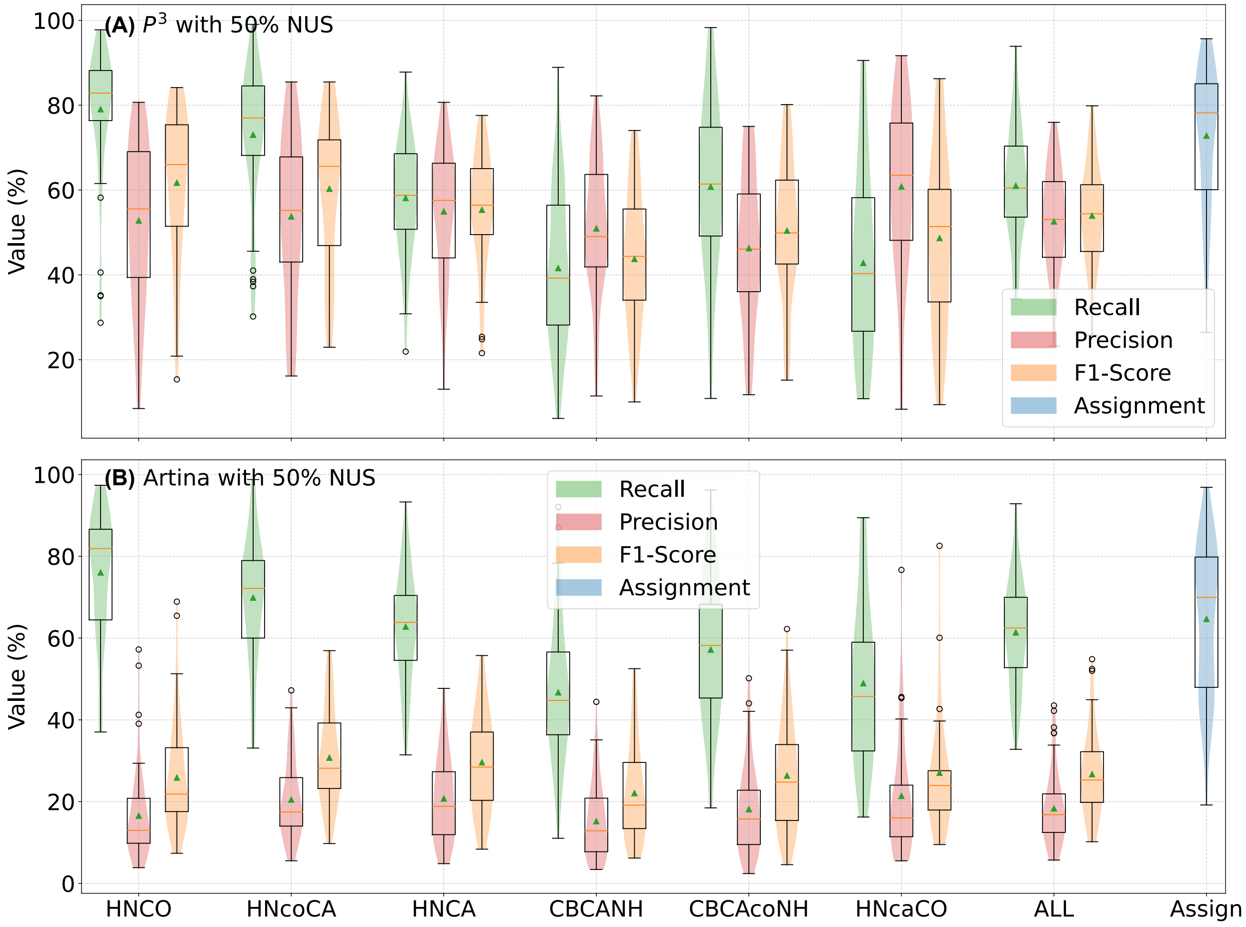

Figure S26: **Peak-picking and assignment performance at 50% NUS across all 60 proteins.** P^3 retains high precision and F1-scores comparable to the fully sampled case, and CYANA assignments remain accurate across proteins and experiment types. ARTINA shows partial improvement compared to lower NUS levels but still exhibits notable reductions in precision and F1-score. MR-Ai therefore provides more reliable peak lists for downstream analysis even at moderate undersampling.

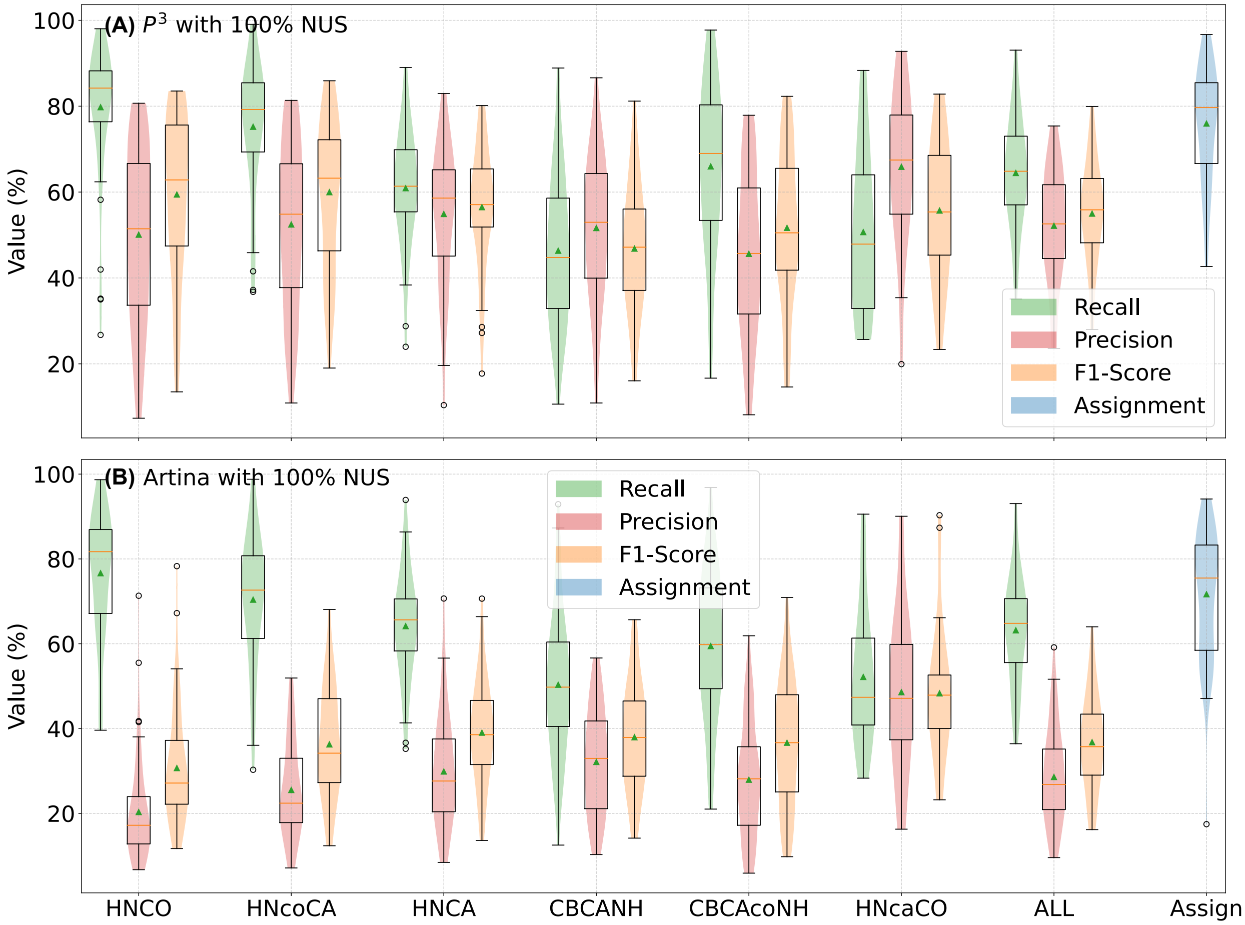

Figure S27: **Peak-picking and assignment performance for fully sampled (100% NUS) spectra across all 60 proteins.** Both methods achieve high recall, reflecting the strong signal quality of fully sampled data. However, P^3 delivers consistently higher precision and F1-scores and results in more accurate CYANA assignments across all six experiment types. This demonstrates that the probabilistic P^3 representation provides advantages even when applied to high-quality fully sampled spectra.

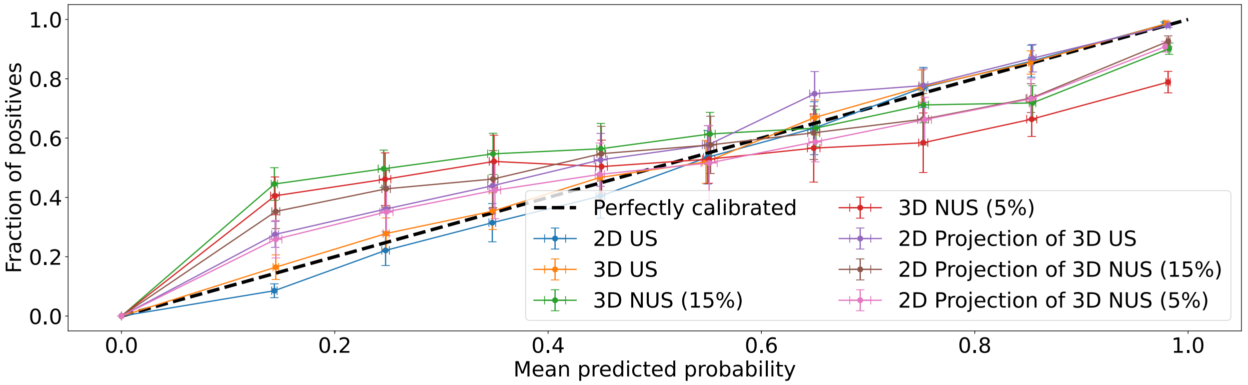

Figure S28: **Statistics on Peak-based probability calibration of P^3 in Synthetic 2D and 3D Spectra.** Predicted probabilities are averaged within bins spanning $0.1 - 0.2$, $0.2 - 0.3$, etc. For each bin, the true fraction of the correct classifications is shown versus the averaged predicted probability. The ideal calibration curve is shown by the diagonal dashed line.

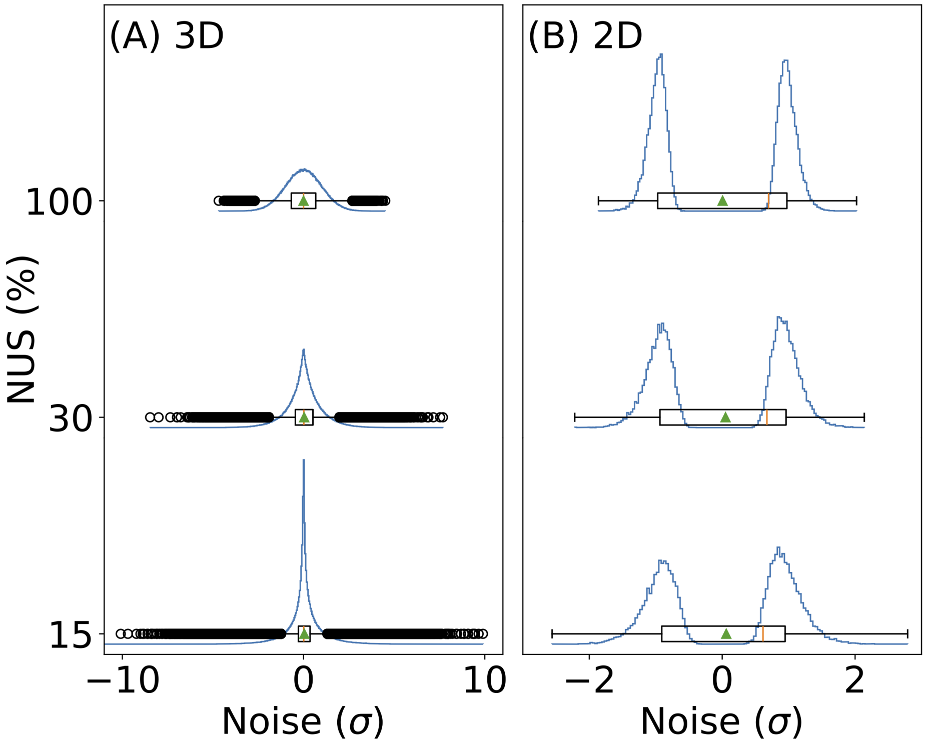

Figure S29: **Distribution of Noise and NUS Aliasing Artifact in synthetic 3D spectra and the 2D Sky projections.** The histograms (blue lines) and Box-plots for the baseline noise are shown for **(A)** synthetic 3D spectra sampled in full (US) and reconstructed using CS-IST from 15% and 30%, NUS. Note the broad Cauchy-like distribution with wide spread of outliers shown in the box-plot for NUS spectra **(B)** 2D skyline projections from the 3D spectra.

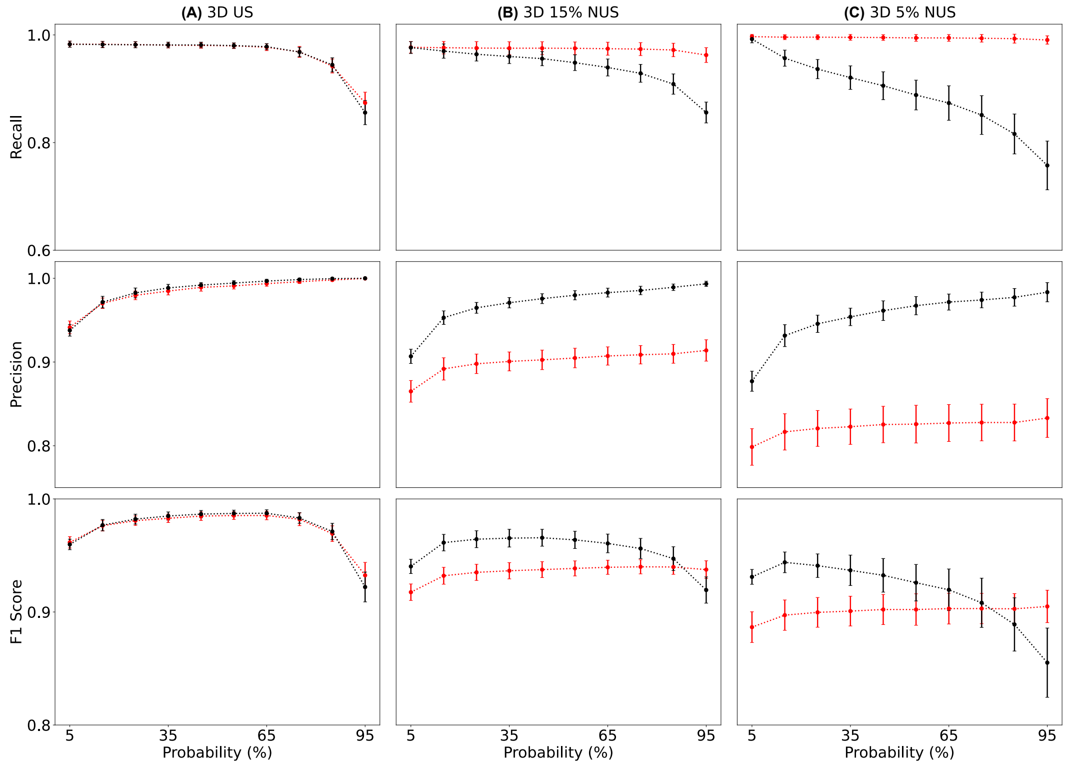

Figure S30: **Comparison of Peak Detection P^3 in Synthetic 3D Spectra Between Two MR-Ai Models Trained with Different Noise Distributions on Training Dataset.** Black and red lines with error bars represent P^3 results from MR-Ai trained on Cauchy-Gaussian and Gaussian noise distributions, respectively, across 10 synthetic NMR spectra, each containing 256 peaks, for **(A)** 3D US, **(B)** 3D 15% NUS reconstructed using CS-IST and **(C)** 3D 15% NUS reconstructed using CS-IST. F1 score is defined as the harmonic mean of the precision and recall scores with $F1 = 2\frac{Precision \times Recall}{Precision + Recall}$. Recall is defined as the ratio of the correctly *detected* pixels to all *detectable* pixels, while precision is the ratio of correctly *detected* pixels to all *detected* pixels. A pixel is considered as *detected* when its P^3 value is above the probability threshold indicated on the horizontal axis of the chart. A pixel is correctly *detected* if it is found in the vicinity of a *detectable* pixel. The *detectable* pixels are defined as those near the maxima of the ground truth peaks with intensities higher than 2σ-noise for the US spectra. To account for the shorter experiment time in the 15% NUS spectra, a threshold of 5σ-noise from the corresponding US spectra was used.

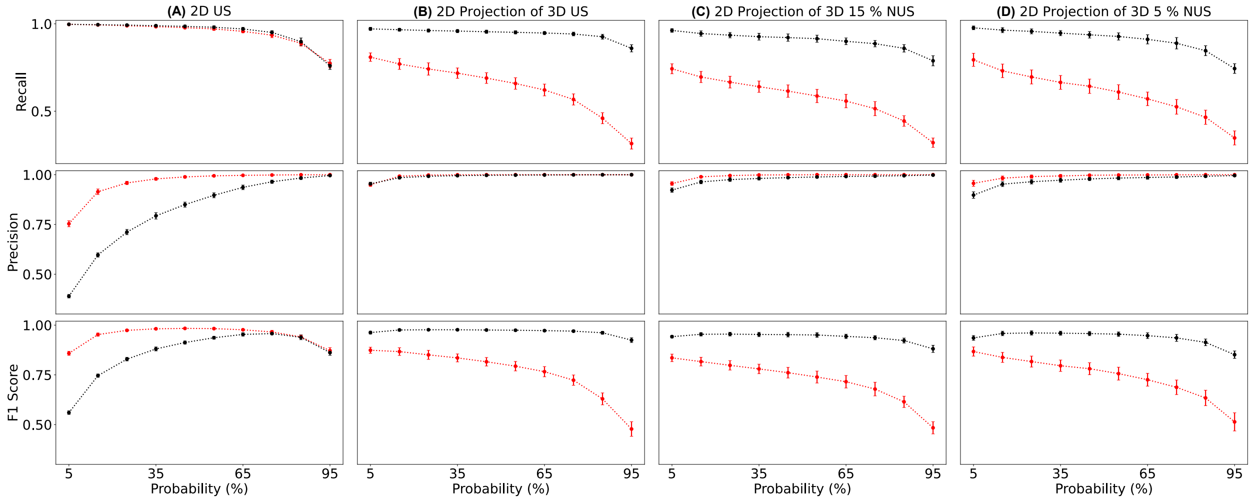

Figure S31: **Comparison of Peak Detection P^3 in Synthetic 2D Spectra Between Two MR-Ai Models Trained with Different Noise Distributions on Training Dataset.** Black and red lines with error bars represent P^3 results from MR-Ai trained on double Gaussian and normal Gaussian noise distributions, respectively, across 10 synthetic NMR spectra, each containing 256 peaks, for **(A)** 2D US, **(B)** 2D projection of 3D US, **(C)** 2D projection of 3D 15% NUS reconstructed using CS-IST, and **(D)** 2D projection of 3D 5% NUS reconstructed using CS-IST. F1 score is defined as the harmonic mean of the precision and recall scores with $F1 = 2\frac{Precision \times Recall}{Precision + Recall}$. Recall is defined as the ratio of the correctly *detected* pixels to all *detectable* pixels, while precision is the ratio of correctly *detected* pixels to all *detected* pixels. A pixel is considered as *detected* when its P^3 value is above the probability threshold indicated on the horizontal axis of the chart. A pixel is correctly *detected* if it is found in the vicinity of a *detectable* pixel. The *detectable* pixels are defined as those near the maxima of the ground truth peaks with intensities higher than 2σ-noise for the US spectra. To account for the shorter experiment time in the 15% NUS spectra, a threshold of 5σ-noise from the corresponding US spectra was used.

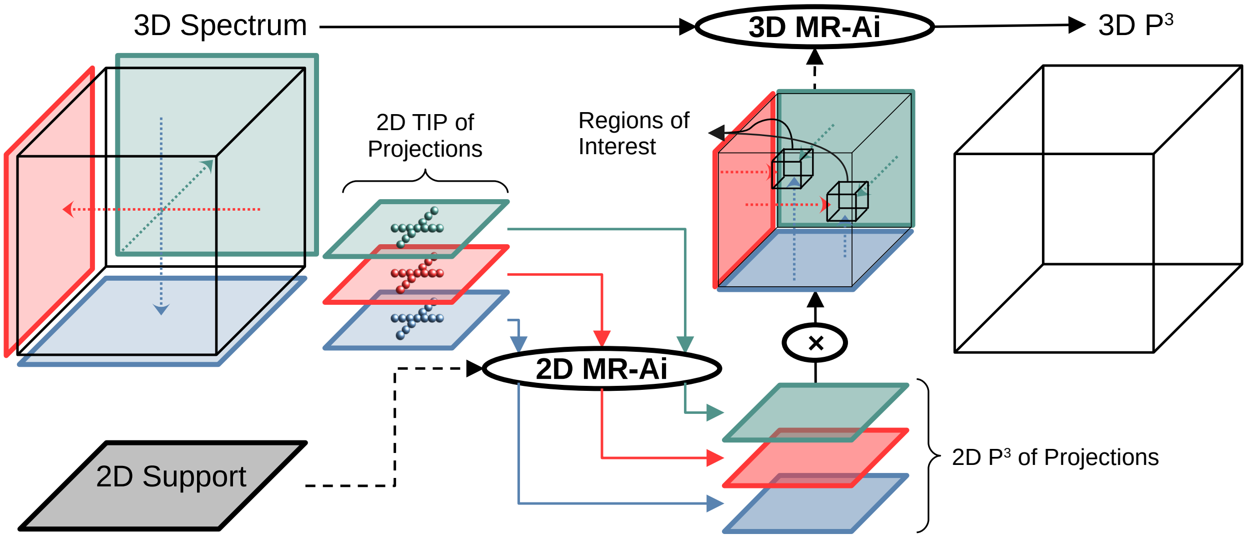

Figure S32: **Schematic of production of 3D P^3 with MR-Ai.** The input 3D spectrum is evaluated by the 3D version of MR-Ai at the points from the regions of interest, which are obtained from the P^3 of the 2D projections and the supporting spectrum (spectra).

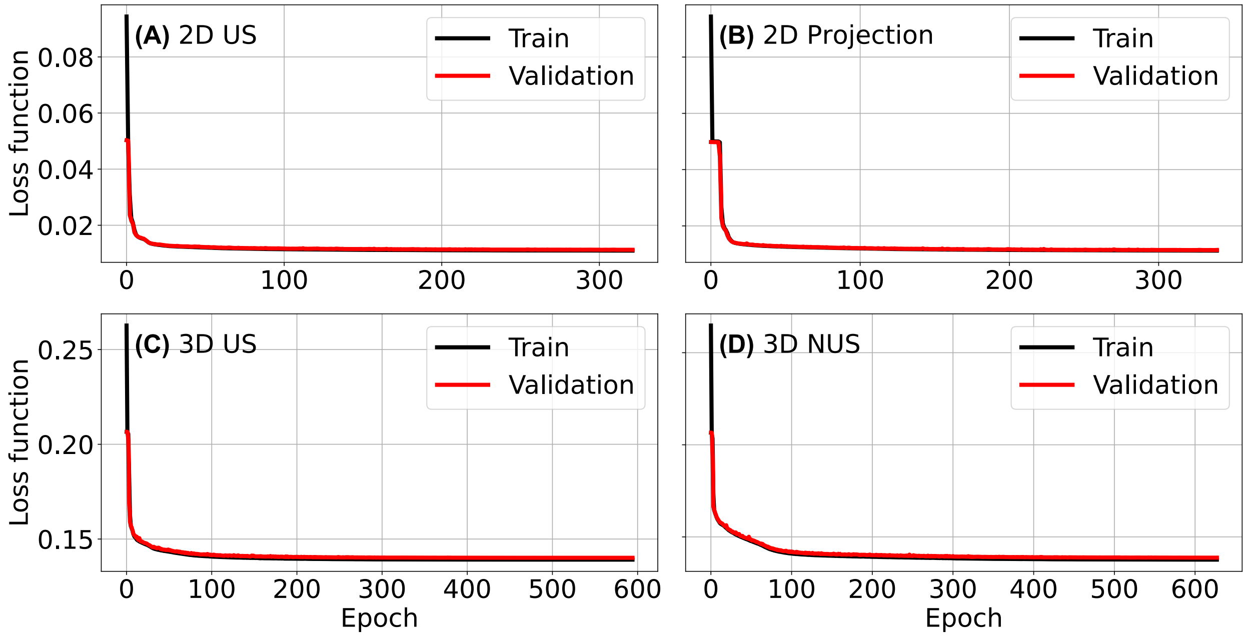

Figure S33: **Convergence of the BCE and cross-validation losses during the MR-Ai P^3 models training.** The curves of training (black) and cross-validation (red) losses for: **(A)** 2D US, **(B)** 2D projection of 3D, **(C)** 3D US, and **(D)** 3D NUS reconstructed spectra. BCE loss below 0.2 indicates good quality for the model probability distribution.
